# A perspective on interaction effects in genetic association studies

**DOI:** 10.1002/gepi.21989

**Published:** 2016-07-07

**Authors:** Hugues Aschard

**Affiliations:** ^1^ Department of EpidemiologyHarvard T.H. School of Public HealthBostonMassachusettsUnited States of America

**Keywords:** genetic risk score, GWAS, interaction, joint test, multivariate analysis, power, pratt index, statistical method, variance explained

## Abstract

The identification of gene–gene and gene–environment interaction in human traits and diseases is an active area of research that generates high expectation, and most often lead to high disappointment. This is partly explained by a misunderstanding of the inherent characteristics of standard regression‐based interaction analyses. Here, I revisit and untangle major theoretical aspects of interaction tests in the special case of linear regression; in particular, I discuss variables coding scheme, interpretation of effect estimate, statistical power, and estimation of variance explained in regard of various hypothetical interaction patterns. Linking this components it appears first that the simplest biological interaction models—in which the magnitude of a genetic effect depends on a common exposure—are among the most difficult to identify. Second, I highlight the demerit of the current strategy to evaluate the contribution of interaction effects to the variance of quantitative outcomes and argue for the use of new approaches to overcome this issue. Finally, I explore the advantages and limitations of multivariate interaction models, when testing for interaction between multiple SNPs and/or multiple exposures, over univariate approaches. Together, these new insights can be leveraged for future method development and to improve our understanding of the genetic architecture of multifactorial traits.

## 1 INTRODUCTION

Hundreds of studies have searched for gene–gene and gene–environment interaction effects in human data with the underlying motivation of identifying or at least accounting for potential biological interaction. So far, this quest has been quite unsuccessful and the large number of methods that have been developed to improve detection (Aschard et al., 2012b; Cordell, 2009; Gauderman, Zhang, Morrison, & Lewinger, 2013; Hutter et al., 2013; Thomas, 2010a; Wei, Hemani, & Haley, 2014) have not qualitatively changed this situation. This lack of discovery in the face of a substantial research investment has been discussed in several review papers that pointed out a number of issues specific to interaction tests, including exposure assessment, time‐dependent effect, confounding effect and multiple comparisons (Aschard et al., 2012b; Bookman et al., 2011; Thomas, 2010b). While these factors are obvious barriers to the identification of interaction effects, it appears that some of the limitations of standard regression‐based interaction tests that pertain to the nature of interaction effects are underestimated. Previous work showed the detection of some interaction effects requires larger sample sizes than marginal effects for a similar effect size (Aiken, West, & Reno, 1991; Greenland, 1983), however it is not an absolute rule. Understanding the theoretical basis of this lack of power can help us optimizing study design to improve detection of interaction effect in human traits and diseases, and open the path for new methods development. Moreover the interpretation of effect estimates from interaction models often suffer from various imprecisions. Compared to marginal models, the coding scheme for interacting variables can impact effect estimates and association signals for the main effects (Aiken et al., 1991; Andersen & Skovgaard, 2010). Also, the current strategy to derive the contribution of interaction effects to the variance of an outcome greatly disadvantages interaction effects and are inappropriate when the goal of a study is not prediction but to assess the relative importance of an interaction term from a biological perspective. While alternative approaches exist, they have not so far been considered in genetic association studies. Finally, the development of new pairwise gene–gene and gene–environment interaction tests is reaching some limits, because the number of prior assumptions that can be leveraged to improve power (e.g. gene–environment independence (Piegorsch, Weinberg, & Taylor, 1994) or the presence of marginal genetic effect for interacting variants (Dai, Kooperberg, Leblanc, & Prentice, 2012) is limited when only two predictors are considered. With the exponential increase of available genetic and nongenetic data, the development and application of multivariate interaction tests offer new opportunities to building powerful approaches and moving the field forward.

## 2 METHODS AND RESULTS

### 2.1 Coding scheme and effect estimates

Consider an interaction effect between a single nucleotide polymorphism (SNP) *G* and an exposure *E* (which can be an environmental exposure or another genetic variant) on a quantitative outcome *Y*. For simplicity I assume in all further derivation that *E* is normally distributed with variance 1, and *G* and *E* are independents. The simplest and most commonly assumed underlying model for *Y* when testing for an interaction effect between *G* and *E* is defined as follows:

$$Y=\beta_{G}\times G+\beta_{E}\times E+\beta_{GE}\times G\times E+\varepsilon$$

where $\beta_{G}$ is the main effect of *G*, $\beta_{E}$ is the main effect of *E*, *β*_GE_ is a linear interaction between *G* and *E*, and ε, the residual, is normally distributed with mean and variance σ^2^ set so that *Y* as mean of 0 and variance of 1 (so the absence of the intercept term in the above equation). One can then evaluate the impact of applying linear transformation of the genotype and/or the exposure when testing for main and interaction effects. For example, assuming *E* has a mean > 0 and *G* is defined as the number of coded allele in the generative model, *Y* can be rewritten as a function of $G_{std}$ and $E_{std}$, the standardized *G* and *E*:

$$Y=\beta_{G}^{\prime }\times G_{std}+\beta_{E}^{\prime }\times E_{std}+\beta_{GE}^{\prime }\times G_{std}\times E_{std}+\varepsilon^{\prime }$$

where $\beta_{G}^{\prime }$, $\beta_{E}^{\prime }$, and $\beta_{GE}^{\prime }$ are the main effects of $G_{std}$ and $E_{std}$ and their interaction. Relating the standardized and unstandardized equations, we obtain (supplementary Appendix A):

$$\beta_{G}^{\prime }=\left(\beta_{G}+\beta_{GE}\times \mu_{E}\right)\times \sigma_{G}\;$$

$$\beta_{E}^{\prime }=\left(\beta_{E}+\beta_{GE}\times \mu_{G}\right)\times \sigma_{E}$$

$$\beta_{GE}^{\prime }=\beta_{GE}\times \sigma_{E}\times \sigma_{G}$$

where $\mu_{G}$, $\sigma_{G}$, $\mu_{E}$, and $\sigma_{E}$ are the mean and variance of *G* and *E*, respectively. Hence, the estimated main effects of $G_{std}$ and $E_{std}$ not only scale with the variance of *G* and *E* but can also change qualitatively if there is an interaction effect (i.e. the direction of the effect can change). In comparison, the interaction effect $\beta_{GE}^{\prime }$ remains qualitatively similar, however, because $\beta_{GE}^{\prime }$ does not scale with $\sigma_{GE}$ the variance of the interaction term but with the variance of *G* and *E*, the interpretation of the relative importance of the interaction effect can change (see Section 2.3).

**Figure 1:**
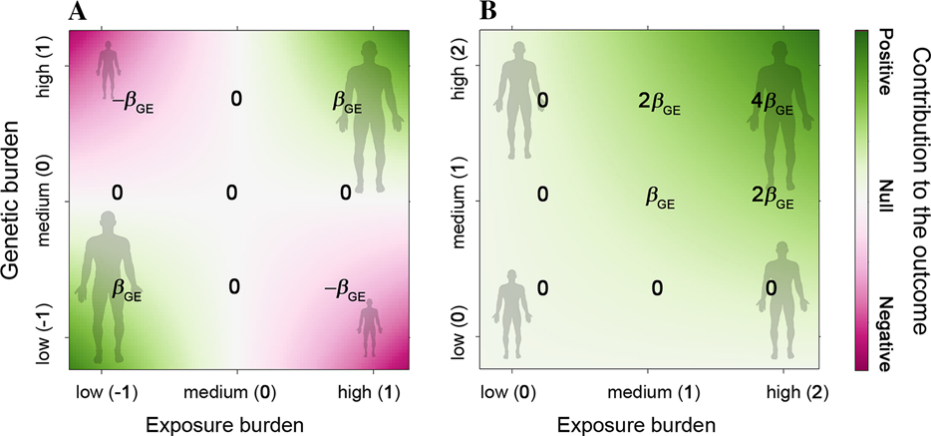
Example of a gene by exposure interaction effect on height. Pattern of contribution of a hypothetical genetic‐by‐exposure interaction term to human height when shifting the location of the genetic and the exposure burdens in the generative model. Genetic burden can correspond to the number of coded allele for a given SNP and exposure burden can correspond to the measure of an environmental exposure. Examples of simple coding are defined in parenthesis on both axes, and the resulting contributions to each specific combination of genetic and environmental values are defined on each panel for a given interaction effect parameter $\beta_{GE}$. In (A) the interaction is defined as the product of a centered genetic variant and a centered exposure. Such encoded interaction induces positive contribution to the outcome for the two extreme groups: (i) maximum exposure burden and maximum genetic burden, and (ii) lowest exposure burden and lowest genetic burden; and negative contribution to the outcome for the two opposite groups: (iii) maximum exposure burden and lowest genetic burden, and (iv) lowest exposure burden and maximum genetic burden. In (B) genetic and exposure burdens are encode in their natural scale and are therefore positive or null. Such coding induces a contribution of the interaction effect to the outcome that is monotonic with increasing genetic and exposure burden.

Which coding scheme for *G* and *E* has the most biological sense can only be discussed on a case by case basis (Aiken et al., 1991). Indeed, defining the optimal coding for a biological question can be very challenging, and as noted in previous work, “*most mathematical models are convenient fictions and would certainly be rejected given sufficient sample size*” (Clayton, 2009). Yet, it is important to recognize that coding scheme should be chosen carefully when testing an interaction as different coding can correspond to qualitatively different relative contribution of each predictor (the main and interaction terms) to the outcome. This is illustrated in Figure 1, which shows the contribution of a pure interaction effect ($\beta_{G}=\beta_{E}=0$ and $\beta_{GE}\neq 0$) to *Y*. When *G* and *E* are centered, the interaction term has a positive contribution to the most extreme subgroups (low exposure and homozygote for the protective allele vs. high exposure and homozygote for the risk allele) and a negative contribution to the opposite heterogeneous subgroups (low exposure and homozygote for the risk allele vs. high exposure and homozygote for the protective allele, Fig. 1A). Conversely, when *G* and *E* are positive or null only, the interaction term corresponds to a monotonic increase (or decrease if the interaction effect is opposite to the main effects) of the magnitude of the genetic and environmental effects (Fig. 1B). Hence, allowing *G* and/or *E* to have negative values in the generative model implies an interaction effect that could be difficult to interpret from a biological perspective. Furthermore, one can easily show that when the mean of the exposure increases while its variance is fixed (e.g. if an environmental exposure increases affect the entire population), an interaction effect will appear more and more as a sole genetic effect (supplementary Fig. S1). Overall, coding schemes that can be related through linear transformations are mathematical equivalent (they produce the same outcome values as long as the predictor effects are rederived to account for the transformation). However, coding scheme should not be overlooked because of this equivalence, and as shown in the example of Figure 1, variable coding should be justified whenever the interpretation of effect estimates matters.

Hopefully, the choice of a specific coding scheme, how to interpret effect estimates when modeling an interaction, and the motivation for adding nonlinear terms in general have been already debated, and several general guidelines have been proposed (see for example the review by Robert J. Friedrich (Friedrich, 1982)). The consensus is that, if the range of the independent variables do naturally includes zero (e.g. smoking status, genetic variants) there is no problem in interpreting the estimated main and interaction effect. For an interaction effect between A and B, the main effect of A corresponds to the effect of A when B is absent and conversely. On the contrary, if the range of the variables do not naturally encompass zero, then the observed estimates “*will be an extrapolations beyond the observed range of experience”* (Friedrich, 1982). Centering the variables can be an option to address this concern. In that case, the main effect of A and B would represent the effect of A among individuals having the mean value of B and conversely. However, as mentioned previously, using centered variables induces a less interpretable interaction term. I suggest that a reasonable alternative would consists in shifting the exposure values so that it has a minimum value close to 0, or alternatively to use ordinal categories of the exposure (e.g. high vs. low BMI as done to define obesity), so that the main effect of A would correspond to the effect among the lowest observed value of B in the population and conversely.

### 2.2 Power considerations

The power of the tests from the interaction model and from a marginal genetic model defined as $Y=\beta_{mG}\times G+\varepsilon_{m}$, can be compared when deriving the noncentrality parameters (*ncp*) of the predictors of interest. Assuming all effects are small, so that σ^2^ the residual variance is close to 1, these *ncps* can be approximated by (see supplementary Appendix B):

$$ncp_{G}\approx N\times \sigma_{G}^{2}\times \beta_{G}^{2}\times \frac{\sigma_{E}^{2}}{\mu_{E}^{2}+\sigma_{E}^{2}}$$

$$ncp_{E}\approx N\times \sigma_{E}^{2}\times \beta_{E}^{2}\times \frac{\sigma_{G}^{2}}{\mu_{G}^{2}+\sigma_{G}^{2}}$$

$$ncp_{GE}\approx N\times \sigma_{E}^{2}\times \sigma_{G}^{2}\times \beta_{GE}^{2}=N\times \beta_{GE}^{{}^{\prime }2}$$

$$ncp_{mG}\approx N\times \sigma_{G}^{2}\times {\left(\beta_{G}+\beta_{GE}\times \mu_{E}\right)}^{2}=N\times \beta_{G}^{{}^{\prime }2}$$

Note that in such scenario adjusting for the effect of *E* in the marginal genetic model has a minor impact on $ncp_{mG}$.

**Figure 2:**
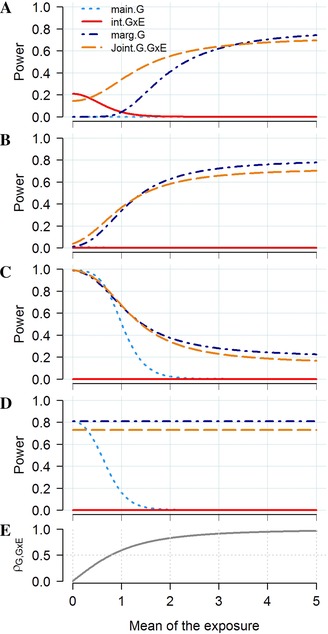
Relative power of the joint test of main genetic and interaction effects. Power comparison for the tests of the main genetic effect (*main.G*), the interaction effect (*int.GxE*), and the joint effect (*Joint G.GxE*) from the interaction model, and the test of the marginal genetic effect (*mar.G*). The outcome *Y* is define as a function of a genetic variant *G* coded as [0,1,2] with a minor allele frequency of 0.3, and the interaction of *G* with an exposure *E* normally distributed with variance 1 and mean $\bar{E}$. The genetic and interaction effects vary so that they explain 0% and 0.04% (A), 0.1% and 0.1% (B), 0.6% and 0.1% (C) with effect in opposite direction, and 0.4% and 0% (D) of the variance of *Y*, respectively. Power and $\rho_{G,G\times E}$, the correlation between *G* and the G×E interaction term (E) were plotted for a sample size of 10,000 individuals and increasing $\bar{E}$ from 0 to 5.

The above equations indicate first that the significance of the marginal test of *G* ($ncp_{mG}$) and the interaction test ($ncp_{GE}$) are invariant with the coding used in the model tested, while the significance of the test of the main genetic and exposure effects can change dramatically when shifting the mean of *G* and *E*. Second, as illustrated in Figure 2, depending on the parameters of the distribution of the exposure and the genetic variants in the generative model, the relative power of each test can be dramatically different. For example, if the genetic variant has only a main linear effect but is not interacting with the exposure, we obtain $ncp_{G}=ncp_{mG}\times \sigma_{E}^{2}/\left(\mu_{E}^{2}+\sigma_{E}^{2}\right)$, so that testing for $\beta_{mG}$ will be much more powerful that testing for $\beta_{G}$ if the mean of *E* is large, although there is no interaction effect here. When the generative model includes an interaction effect only ($\beta_{G}=\beta_{E}=0$ and $\beta_{GE}\neq 0$), we obtain $ncp_{mG}=ncp_{GE}\times \mu_{E}^{2}/\sigma_{E}^{2}$. Again, the marginal test of the genetic effect can be dramatically more powerful than the test of interaction effect although the generative model includes only an interaction term but no main effect.

It follows that the power to detect an interaction effect explaining for example 1% of the variance of *Y* (where variance explained for a predictor *X* is defined as $\beta_{X}^{2}/\sigma_{X}^{2}$) but inducing no marginal genetic effect (i.e. when *E* is centered as in Fig. 1A) is much higher than for an interaction explaining the same amount of variance but whose effect can be capture by a marginal term (i.e. when *E* is not centered as in Fig. 1B–D). This result is a direct consequence of the covariance between $\beta_{G}$ and $\beta_{GE}$ that arise when having noncentered exposure in the generative model (Fig. 2E). This covariance equals $\mu_{E}\times \sigma_{G}^{2}$ (supplementary Appendix C). It induces uncertainty on the estimation of the predictor effects, which decreases the significance of the estimates in the interaction model. With increasing intercorrelations between predictors it becomes impossible to disentangle the effects of one predictor from another, the standard errors of the effect estimates becoming infinitely large and the power decreases to the null (Farrar & Glauber, 1967). As showed in the simulation study from supplementary Figures S2 and S3 these results appear consistent for both linear and logistic regression and when assuming non‐normal distribution of the exposure.

This lead to the nonintuitive situation where the power to detect a relatively simple and parsimonious interaction effect from a biological perspective–defined as the product of a genetic variant and an exposure both coded to be positive or null–is very small; and in most scenarios where the main genetic and interaction effects do not canceled each other (see e.g. Weiss, 2008) the marginal association test of *G* would be more powerful. In comparison a more exotic interaction effect as defined in Figure 1A and supplementary Figure S1E, would be both much easier to detect and not captured in a screening of marginal genetic effect.

### 2.3 Proportion of variance explained

In genetic association studies the proportion of variance explained by an interaction term is commonly evaluated as the amount of variance of the outcome it can explain on top of the marginal linear effect of the interacting factors (Hill, Goddard, & Visscher, 2008). Following the aforementioned principle, one can derive the contribution of *G* ($r_{G}^{2}$), *E* ($r_{E}^{2}$), and G×E ($r_{GE}^{2}$) to the variance of the outcome using the estimates from the standardize model, in which the interaction term is independent from *G* and *E* (supplementary Appendix D):

$$r_{G}^{2}=\beta_{G}^{{}^{\prime }2}={\left(\beta_{G}+\beta_{GE}\times \mu_{E}\right)}^{2}\times \sigma_{G}^{2}$$

$$r_{E}^{2}=\beta_{E}^{{}^{\prime }2}={\left(\beta_{E}+\beta_{GE}\times \mu_{G}\right)}^{2}\times \sigma_{E}^{2}$$

$$r_{GE}^{2}=\beta_{GE}^{{}^{\prime }2}={\left(\beta_{GE}\times \sigma_{E}\times \sigma_{G}\right)}^{2}.$$

**Figure 3:**
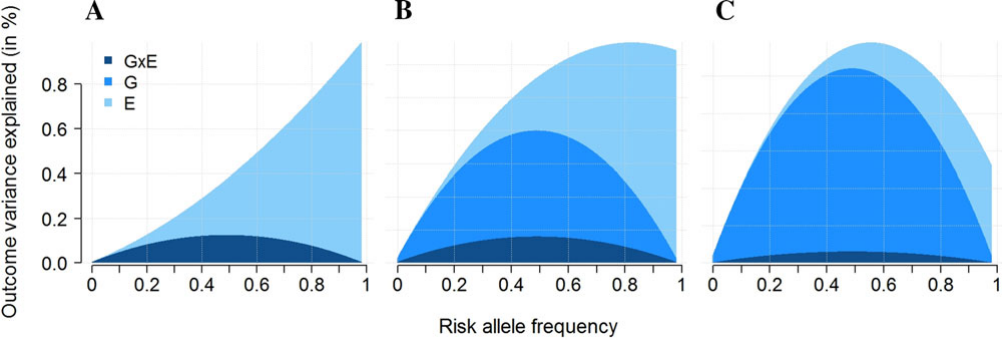
Examples of attribution of phenotypic variance explained by an interaction effect. Proportion of variance of an outcome *Y* explained by a genetic variant *G*, an exposure *E* and their interaction *G* × *E* in a model harboring a pure interaction effect only ($Y=\beta_{GE}\times G\times E+\varepsilon$). The exposure *E* follows a normal distribution with a standard deviation of 1 and mean of 0 (A), 2 (B), and 4 (C). The genetic variant is biallelic with a risk allele frequency increasing from 0.01 to 0.99. The interaction effect is set so that the maximum of the variance explained by the model equals 1%.

The total variance explained by the predictors in the interaction model equals $r_{model}^{2}=r_{G}^{2}+r_{E}^{2}+r_{GE}^{2}$. It follows that one can draw various scenarios where the estimated main effect of *E* and *G* can be equal to zero but have a nonzero contribution to the variance of *Y* because of the interaction effect. Consider the simple example where *G* and *E* are binary variables and have a pure synergistic effect, that is, the effect of *G* and *E* is observed only in the exposed subjects carrying the risk allele. Following the above equations, if *G* and *E* have frequencies of, e.g. 0.3 and 0.7 and $\beta_{GE}=0.5$, the contribution of *G*, *E*, and G×E to the variance of the outcome equal 2.56%, 0.47% and 1.10%, respectively. More generally, Figure 3 shows that depending on the frequency of the causal allele and the distribution of the exposure in the generative model, the vast majority of the contribution of the interaction term to the variance of *Y* will be attributed to either the genetic variant or the exposure. This is in agreement with previous work showing that even if a large proportion of the genetic effect on a given trait is induced by interaction effects, the observed contribution of interaction terms to the heritability can still be very small (Hill et al., 2008). Because such interaction effects have small contribution to $r_{model}^{2}$ on top of the marginal effects of *E* and *G*, they have a very limited utility for prediction purposes in the general population (Aschard et al., 2012a; Aschard, Zaitlen, Lindstrom, & Kraft, 2015).

Still, this is a strong limitation when the goal is not prediction but to understand the underlying architecture of the trait under study and to evaluate the relative importance of main and interaction effects from a public health perspective. Lewontin (Lewontin, 1974) highlighted similar issues, showing that the analysis of causes and the analysis of variance are not necessarily overlapping concepts. His work presents various scenarios where “*the analysis of variance will give a completely erroneous picture of the causative relations between genotype, environment, and phenotype because the particular distribution of genotypes and environments in a given population*.” Since then, a number of theoretical studies have explored the issue of assigning importance to correlated predictors (Budescu, 1993; Chao, Zhao, Kupper, & Nylander‐French, 2008; Darlington, 1968; Green, Carroll, & DeSarbo, 1978) and several alternatives measures have been proposed. To my knowledge, none of these measures has been considered so far in human genetic association studies. The advantages and limitation of these alternative methods have been debated for years and no clear consensus arose, however Pratt axiomatic justification (Pratt, 1987) for one of these method—further presented in the literature as the Product Measure (Bring, 1996), Pratt index or Pratt's measure (Thomas, Hughes, & Zumbo, 1998)—makes it a relevant substitute. For a predictor $X_{i}$, the Pratt's index that we refer further as $r^{2*}$, is defined as the product of $\beta_{X_{i}}$, the standardized coefficient from the multivariate model (where all predictors are scaled to have mean 0 and variance 1, including the interaction term), times its marginal (or zero‐order) correlation with the outcome $cor(Y,X_{i})$, i.e. $r_{X_{i}}^{2*}=\beta_{X_{i}}\times cor\left(Y,X_{i}\right)$.

By definition, $r_{X_{i}}^{2*}$ attributes a predictor's importance as a direct function of its estimated effect and therefore addresses the previously raised concern. Among other relevant properties, it depends only on regression coefficients, multiple correlation, and residual variance but not higher moments, and it does not change with (nonconstant) linear transformation of predictors other than $X_{i}$. It also has convenient additivity properties as it satisfies the condition $r_{G}^{2*}+r_{E}^{2*}+r_{GE}^{2*}=r_{model}^{2}$ (supplementary Appendix D), so that the overall contribution of the predictors is the sum of their individual contribution, and for example the cumulated contribution of multiple interaction effects can easily be evaluated by summing $r_{X_{i}}^{2*}$. The Pratt's index also received criticisms (Bring, 1996; Chao et al., 2008), in particular for allowing $r_{X}^{2*}$ being negative (Thomas et al., 1998). Pratt's answer to this concern is that $r_{X_{i}}^{2*}$ only describes the average contribution of a predictor to the outcome variance in one dimension and is therefore, as any one‐dimension measure, a suboptimal representation of the complexity of the underlying model. For example, a negative $r_{X_{i}}^{2*}$ means that if we were able to remove the effect of $X_{i}$, the variance of the outcome would increase because of the correlation of $X_{i}$ with other predictors (see example from supplementary Appendix D).

From a practical perspective, $r_{X_{i}}^{2*}$ can be expressed as a function of the estimated effects, the means and the variances of *E* and *G* (supplementary Appendix D), and can be derived using estimates from a standard regression model:

$$r_{G}^{2*}=\left(\beta_{G}^{2}+\beta_{G}\times \beta_{GE}\times \mu_{E}\right)\times \sigma_{G}^{2}$$

$$r_{E}^{2*}=\left(\beta_{E}^{2}+\beta_{E}\times \beta_{GE}\times \mu_{G}\right)\times \sigma_{E}^{2}$$

$$\begin{array}{ccc} r_{GE}^{2*} & = & \beta_{GE}^{2}\times \sigma_{GE}^{2}+\beta_{GE}\\ & & \times \left(\beta_{G}\times \mu_{E}\times \sigma_{G}^{2}+\beta_{E}\times \mu_{G}\times \sigma_{E}^{2}\right) \end{array}$$

**Figure 4:**
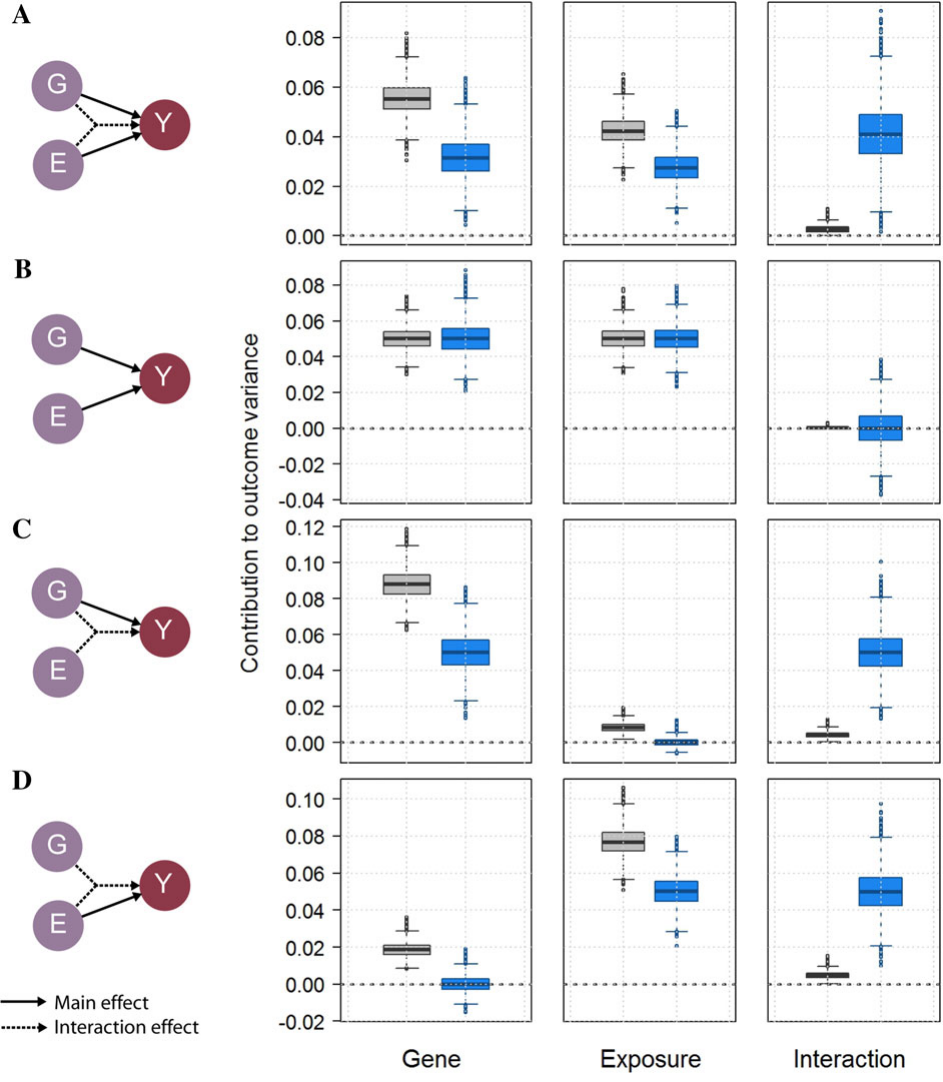
Relative importance of an interaction term as defined by the Pratt index. Contribution of a genetic variant *G* with minor allele frequency of 0.5, a normally distributed exposure *E* with mean of 4 and variance of 1 and their interaction *G* × *E*, to the variance of a normally distributed outcome *Y*, based on the standard approach‐–the marginal contribution of *E* and *G* and the increase in *r* ^2^ when adding the interaction term–(gray boxes), and based on the Pratt index (blue boxes), across 10,000 replicates of 5,000 subjects. For illustration purposes the predictors explain jointly 10% of the variance of *Y*. In scenario (A) all G, E, and G × E have equal contribution, while in scenarios (B), (C), and (D) there is no interaction effect, no exposure effect, and no genetic effect, respectively.

As showed in Figure 4 and supplementary Figure S4, the Pratt index can recover the pattern of the causal model in situations where the standard approach would underestimate the importance of the interaction effects. It can therefore be of great use in future studies to evaluate the importance of potentially modifiable exposures that influence the genetic component of multifactorial traits.

Table 1 illustrates the differences between the two approaches for two confirmed interaction effect on body mass index (BMI). In case (1), the authors identified and replicated an interaction between soda consumption and a genetic risk score (GRS) of 32 BMI SNPs. Case (2) is a replication of a previously identified interaction between a GRS of 12 BMI SNPs and physical activity (Ahmad et al., 2013). Following the formulas above and using approximation of mean and variance of the genetic and exposure variables (supplementary Table S1 and S2), I estimated the contribution of each term using the standard approach and the Pratt index after rederiving effect estimates for a model where predictor values (for the GRS and the exposure) are shifted so that the minimum observed values equal 0—as suggested earlier. It resulted in major differences of the relative importance of the three predictors, the contribution of the interaction effect as derived with the Pratt Index being substantially higher in both cases (increasing from 4.4% to 10.8% for case 1, and from 0.4% to 15.7% for case 2). Case 1 highlights in particular that reducing soda consumption might have a greater impact in reducing the average BMI in the population than one would expect when focusing on the amount of variance explained as defined in the standard approach. An important caveat here is that the Pratt Index is sensitive to location shift of the predictor (as performed in this analysis) and the results from Table 1 would change if a different transformation was applied to the predictors (i.e. if the minimum possible value was defined differently). In comparison, the standard approach is robust to linear transformation of the predictors.

**Table 1:** Relative importance of GRS by exposure interaction effect from real data example

Reference	Contribution to BMI	Standard^a^	Pratt index
32 BMI SNPs × soda consumption	Total	0.011	0.011
	% of genetic	91.0%	70.5%
	% of environment	4.6%	18.7%
	% of interaction	4.4%	10.8%
12 BMI SNPs × physical Activity	Total	0.016	0.016
	% of genetic	43.8%	49.1%
	% of environment	55.8%	35.2%
	% of interaction	0.4%	15.7%

BMI, body mass index; SNP, single nucleotide polymorphisms.

a Variance explained for the interaction effect is derived as the variance explained on top of the marginal contribution from the genes and the environment.

### 2.4 Improving detection through multivariate interaction tests

Using statistical technics such as the Pratt index can provide clues on the importance of interaction effects; however it does not help in mapping interaction. Increasing power mostly relies on two principles: increasing sample size, and leveraging assumptions on the underlying model. The case‐only test, which assumes independence between the genetic variant and the exposure, and a two steps strategy that select candidate variants for interaction test based on their marginal linear effects, are good examples of the later principle (Dai et al., 2012; Gauderman et al., 2013; Mukherjee, Ahn, Gruber, & Chatterjee, 2012). However, only a limited number of assumptions can be made for a single variant by a single exposure interaction test. With the overwhelming wave of genomic and environmental data, I suggest that a major path to move the field forward is to extend this principle while considering jointly more parameters.

This has actually already been applied over the past few years with the joint test of main genetic and interaction effects (Kraft, Yen, Stram, Morrison, & Gauderman, 2007). The *ncp* of such a joint test can be expressed as a function of main and interaction estimates ($\beta_{G}$ and $\beta_{GE}$), their variances ($\sigma_{\beta_{G}}^{2}$ and $\sigma_{\beta_{GE}}^{2}$) and their covariance γ (supplementary Appendix E). By accounting for γ the joint test recovers most of the power lost by the univariate test of the main genetic and interaction effect (so the situation where neither the interaction effect nor the main genetic effect are significant, while the joint test is, see e.g. SNP rs11654749 in (Hancock et al., 2012)). More importantly, in the presence of both main and interaction effects, it can outperform the marginal test of *G*. Although this is at the cost of decreased precision, i.e. if the test is significant, one cannot conclude whether association signal is driven by the main or the interaction effect. Moreover this would be true only if the contribution of the interaction effect on top of the marginal effect is large enough so that it balanced the increase in number of degree of freedom (Aschard, Hancock, London, & Kraft, 2010; Clayton & McKeigue, 2001) (Fig. 2).

Application of the joint test of main genetic effect and a single gene by exposure interaction term is now relatively common in GWAS setting (Hamza et al., 2011; Hancock et al., 2012; Manning et al., 2012). However, exploring further multivariate interactions with multiple exposures is limited by practical considerations. Existing software to perform the joint test in a meta‐analysis context (Aschard et al., 2010; Manning et al., 2011) only allow the analysis of a single interaction term mostly because it requires the variance‐covariance matrix between estimates, which is not provided by popular GWAS software. Leveraging the results from the previous sections on can show that the *ncp* of the joint test of main genetic effect and interactions with *l* independent exposures can be expressed as the sum of *ncp* from the test of *G* and the $G\times E_{cent.i}$ where $E_{cent.i}$ is the centered exposure *i* (supplementary Appendix E):

$$ncp_{G+GE}=N\times \sigma_{G}^{2}\times \beta_{G}^{{}^{\prime \prime }2}+\sum_{i=1...l}\left[N\times \sigma_{G}^{2}\times \sigma_{E}^{2}\times \beta_{GE_{cent.i}}^{{}^{\prime \prime }2}\right]$$

where $\beta_{G}^{{}^{\prime \prime }}$ and $\beta_{GE_{cent.i}}^{{}^{\prime \prime }}$ are the effects of *G* and $G\times E_{cent.i}$. Such a test is robust to non‐normal distribution of the exposure, and modest correlation (<0.1) between the genetic variant and the exposures, but sensitive to moderate correlation (>0.1) between exposures (supplementary Figs. S5 and S6). Hence, one can perform meta‐analysis of a joint test including multiple interaction effects using existing software simply by centering exposures. In brief one would have to perform first a standard inverse‐variance meta‐analysis to derive chi‐squares for the l+1 terms from the model considered, and then to sum all chi‐squares to form a chi‐square with l+1 *df*. Importantly, centering the exposures will be of interest only when testing jointly multiple interactions and the main genetic effect. In comparison, the combined test of multiple interaction effects can be simply performed by summing chi‐squares from each independent interaction test or from interaction test derived in a joint model. As previously, the validity of this approach relies on independence between the genetic variant and the exposures, and between the exposures. Finally, a more general solutions that should be explored in future studies would consists, as proposed for the analysis of multiple phenotypes (e.g. Zhu et al., 2015), in estimating the correlation between all tests considered (main genetic effect and/or multiple interaction effects) using genome‐wide summary statistics in order to form a multivariate test.

**Figure 5:**
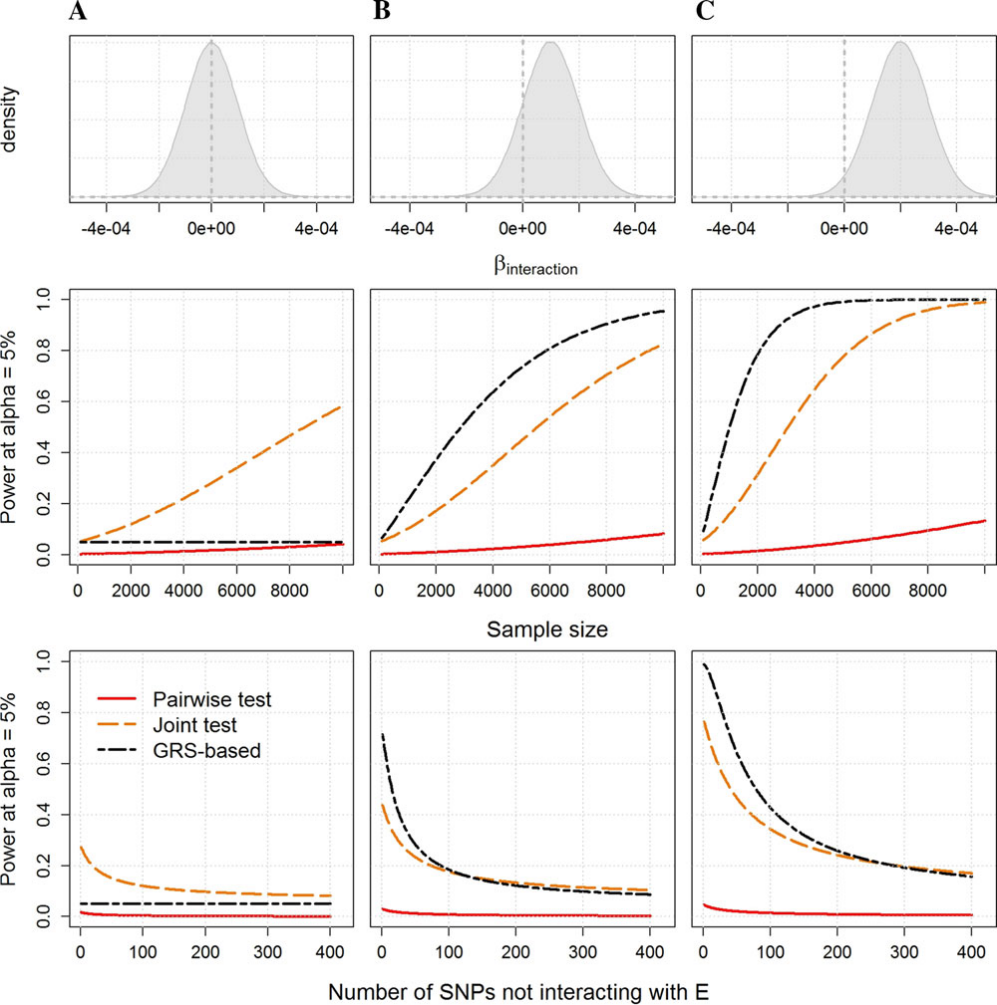
Advantages and limitations of testing interaction effect with a genetic risk score. Examples of power comparison for the combined analysis of interaction effects between 20 SNPs and a single exposure. Power was derived for three scenarios: the interaction effects are normally distributed (upper panels) and (A) centered, (B) slightly positive so that 25% of the interactions are negative, and (C) positive only. Three tests are compared while increasing sample size from 0 to 10,000: the joint test of all interaction terms, the genetic risk score by exposure interaction test, and the test of the strongest interaction effect (pairwise test) after correction for the 20 tests performed (middle panels). The lower panels show power of the three tests for a sample size of 5,000, when including 1–400 non‐interacting SNPs on top of the 20 causal SNPs in the analysis and after accounting for multiple testing in the pairwise test.

A second major direction for the development of multivariate test is to assume the effects of multiple genetic variants depend on a single “scaling” variable *E*. A rising approach consists in testing for interaction between the scaling variable and a genetic risk score (GRS), derived as the weighted sum of the risk alleles. Several interaction effects have been identified using this strategy (Ahmad et al., 2013; Fu et al., 2013; Langenberg et al., 2014; Pollin et al., 2012; Qi, Cornelis, Zhang, van Dam, & Hu, 2009; Qi et al., 2012), some being replicated in independent studies (Ahmad et al., 2013; Qi et al., 2012). This relative success, as compared to univariate analysis, has generated discussion regarding potential underlying mechanisms (Aschard et al., 2015; Ebbeling & Ludwig, 2013; Goran, 2013; Greenfield, Samaras, & Campbell, 2013; Malavazos, Briganti, & Morricone, 2013). Overall, testing for an interaction effect between a GRS and a single exposure consists in expanding the principle of a joint test of multiple interactions while leveraging the assumption that, for a given choice of coded alleles, most interaction effects are going in the same direction. It is similar in essence to the burden test that has been widely used for rare variant analysis (Lee, Abecasis, Boehnke, & Lin, 2014). In its simplest form it can be expressed as the sum of all interaction effects and it captures therefore deviation of the mean of interaction effects from 0. When interaction effects are null on average, a joint test of all interaction tests (as previously described) will likely be the most powerful approach as it allows interaction effects to be heterogeneous. Conversely, if interactions tend to go in the same direction, the GRS‐based test can outperform other approaches (Fig. 5 **)**. Of course, in a realistic scenario, a number of non‐interacting SNPs would be included in the GRS, diluting the overall interaction signal and therefore decreasing power. However, the gain in power for the multivariate approaches can remain substantial even when a large proportion of the SNPs tested (e.g. 95% in the example from Fig. 5) is not interacting with the exposure. Table 2 illustrates power achieved by these tests in the examples used for Table 1.

**Table 2:** Genetic risk score by exposure interaction in real data

		32 BMI SNP × soda consumption	12 BMI SNP × physical activity
Reported *P*‐value	*Best SNP* a	0.0030	0.0030
	*GRS from paper* c	<0.001	0.016
Derived *P*‐value^a^	*wGRS*	0.000028	0.0027
	*uGRS*	0.00019	0.015
	*chi2*	0.014	0.050
Power^b^	*Best SNP*	0.43	0.68
	*wGRS*	0.99	0.85
	*uGRS*	0.96	0.54

SNP, single nucleotide polymorphisms; wGRS, weighted GRS; uGRS, unweighted GRS; chi2, sum of individual interaction chi‐squared.

a *P*‐values derived from individual SNP by exposure interaction estimates, not corrected for the number of SNPs tested.

b Power is approximated based on the effect estimate. It is derived for an alpha level of 5% and sample sizes similar to those used in the corresponding study.

c For soda consumption, the authors used a weighted GRS, for physical activity, the authors used an unweighted GRS.

Finally, as showed in supplementary Appendix F, assuming the SNPs in the GRS are independents from each others, the GRS by *E* interaction test can be derived from individual interaction effect estimates. More precisely, consider testing the effect of a weighted GRS on *Y*:

$$Y∼\gamma_{GRS}\times GRS+\gamma_{E}\times E+\gamma_{INT}\times GRS\times E$$

where $\gamma_{GRS}$, $\gamma_{E}$, and $\gamma_{INT}$ are the main effect of the weighted GRS, the main effect of *E* and the interaction effect between *E* and the GRS, respectively. The test of $\gamma_{INT}$ is asymptotically equivalent to the meta‐analysis of $\gamma_{G_{i}\times E}$, the interaction effects between $G_{i}$ and *E*, using an inverse‐variance weighted sum to derive a 1 *df* chi‐square, i.e. (see supplementary Appendix F, supplementary Figs. S7 and S8, and Dastani et al., 2012):

$${\left(\frac{{\hat{\gamma }}_{INT}}{{\hat{\sigma }}_{\gamma_{INT}}}\right)}^{2}=\frac{{\left(\sum_{m}\frac{w_{i}\times {\hat{\gamma }}_{G_{i}\times E}}{{\hat{\sigma }}_{\gamma_{G_{i}\times E}}^{2}}\right)}^{2}}{\sum_{m}\frac{w_{i}^{2}}{{\hat{\sigma }}_{\gamma_{G_{i}\times E}}^{2}}}\;$$

where $w_{i}$ is the weight given to SNP *i*.

A number of strategies can be used for the weighting scheme. Assuming equal effect size of all interaction effects, one should weight each SNP by the inverse of their standard deviation ($w_{i}=1/\sigma_{G_{i}}$). Alternatively, others have used weights proportional to the marginal genetic effect of the SNPs, assuming the magnitude of the marginal and interaction effects are correlated. Obviously, the relative power of each of these weighting schemes depends on their relevance in regard to the true underlying model. Finally, applying GRS‐based interaction tests implicitly supposed a set of candidate genetic variants have been identified. The current rationale consists in assuming that most interacting variants also display a marginal linear effect and therefore have focused on GWAS hits, however other screening methods can be used (Aschard, Zaitlen, Tamimi, Lindstrom, & Kraft, 2013; Pare, Cook, Ridker, & Chasman, 2010). Moreover, existing knowledge, such as functional annotation (Consortium, 2004) or existing pathway database (Kanehisa et al., 2014) can be leverage to refine the sets of SNPs to be aggregated into a GRS.

## 3 DISCUSSION

Advancing knowledge of how genetic and environmental factors combine to influence human traits and diseases remains a key objective of research in human genetics. Ironically, the simplest and most parsimonious biological interaction models—those in which the effect of a genetic variant is either enhanced or decreased depending on a common exposure—are probably the most difficult to identify. Furthermore, the contribution of such interaction effects can be dramatically underestimated when measured as the drop in *r* ^2^ if the interaction term was removed from the model. Here, I argue for the use of new approaches and analytical strategies to address these concerns. This includes using methods such as the Pratt index to evaluate the relative importance of interaction effects in genetic association studies. These methods can highlight important modifiable exposures influencing genetic mechanisms, which could be neglected with the existing approach. Regarding detection, and besides increasing sample size, increasing power to detect interaction effects in future studies will likely mostly rely on leveraging additional assumptions on the underlying model. In the big data era, where millions of genetic variants are measured on behalf of multiple environmental exposures and endo‐phenotypes, this means using multivariate models. A variety of powerful statistical tests can be devised assuming multiple environmental exposures interact with multiple genetic variants. As showed in this study, the application of such approaches can dramatically improve power to detect interaction that can be missed by standard univariate tests. While these methods comes at the cost of decreased precision—i.e. a significant signal would point out multiple potential culprit—they can identify interaction effects that would potentially be of greater clinical relevance that univariate pairwise interaction (Aschard et al., 2012a, 2015; Qi et al., 2012).

Understanding the strengths and limitations of standard statistical methods is a major key to overcome today's challenges for the identification of interaction effects in human traits and diseases. By deciphering the basic principles of interaction tests, this perspective aims at providing a comprehensive guideline for performing interaction effects analyses in genetic association studies, and opening the path for future method development.

## Supporting information

Additional Supporting Information may be found online in the supporting information tab for this article.

Appendix A: Effect estimates from standardized and unstandardized predictors
Appendix B: Non‐centrality parameters for marginal and interaction models
Appendix C: Variance‐covariance for the GxE term and its estimated effect
Appendix D: Derivation of the Pratt index
Appendix E: Joint test of main and interaction effects
Appendix F: GRS‐based test, joint test and univariate test of multiple interaction effects
Figure S1. Linear interaction effect across different coding schemes
Figure S2. Power comparison for linear regression
Figure S3. Power comparison for logistic regression
Figure S4. The Pratt index across multiple interactions
Figure S5. Joint test of main genetic effect and multiple interaction effects in a linear regression
Figure S6. Joint test of main genetic effect and multiple interaction effects in a logistic regression
Figure S7. GRS‐based statistic and meta‐analysis of single SNP estimates in linear regression
Figure S8. GRS‐based statistic and meta‐analysis of single SNP estimates in logistic regression
