# Supplementary material for: A perspective on interaction effects in genetic association studies
Source: Genet Epidemiol. 2016 Jul 7;40(8):678–88. doi: 10.1002/gepi.21989 (PMC5132101; doi:10.1002/gepi.21989)
Supplement: Supplementary file 1 — Appendix A: Effect estimates from standardized and unstandardized predictors Appendix B: Non‐centrality parameters for marginal and interaction models Appendix C: Variance‐covariance for the GxE term and its estimated effect Appendix D: Derivation of the Pratt index Appendix E: Joint test of main and interaction effects Appendix F: GRS‐based test, joint test and univariate test of multiple interaction effects Figure S1. Linear interaction effect across different coding schemes Figure S2. Power comparison for linear regression Figure S3. Power comparison for logistic regression Figure S4. The Pratt index across multiple interactions Figure S5. Joint test of main genetic effect and multiple interaction effects in a linear regression Figure S6. Joint test of main genetic effect and multiple interaction effects in a logistic regression Figure S7. GRS‐based statistic and meta‐analysis of single SNP estimates in linear regression Figure S8. GRS‐based statistic and meta‐analysis of single SNP estimates in logistic regression [file GEPI-40-678-s001.pdf]

A perspective on interaction effects in genetic association studies

Supporting Information

Contents

Appendix A: Effect estimates from standardized and unstandardized predictors 2

Appendix B: Non-centrality parameters for marginal and interaction models 3

Appendix C: Variance-covariance for the GxE term and its estimated effect 3

Appendix D: Derivation of the Pratt index 5

Appendix E: Joint test of main and interaction effects 8

Appendix F: GRS-based test, joint test and univariate test of multiple interaction effects 7

Figure S1. Linear interaction effect across different coding schemes 8

Figure S2. Power comparison for linear regression 11

Figure S3. Power comparison for logistic regression 12

Figure S4. The Pratt index across multiple interactions. 13

Figure S5. Joint test of main genetic effect and multiple interaction effects in a linear regression. 14

Figure S6. Joint test of main genetic effect and multiple interaction effects in a logistic regression. 15

Figure S7. GRS-based statistic and meta-analysis of single SNP estimates in linear regression. 16

Figure S8. GRS-based statistic and meta-analysis of single SNP estimates in logistic regression. 17

Appendix A: Effect estimates from standardized and unstandardized predictors

Following the notation defined in the main text, the (standardized) outcome Y can be expressed as:

$$\begin{aligned} Y &= \beta_G \times G + \beta_E \times E + \beta_{GE} \times G \times E + \varepsilon \\ &= \beta_G \times (G_{std}\sigma_G + \mu_G) + \beta_E \times (E_{std}\sigma_E + \mu_E) + \beta_{GE} \times (G_{std}\sigma_G + \mu_G) \times (E_{std}\sigma_E + \mu_E) + \varepsilon \\ &= (\beta_G\sigma_G + \beta_{GE}\mu_E\sigma_G) \times G_{std} + (\beta_E\sigma_E + \beta_{GE}\mu_G\sigma_E) \times E_{std} + (\beta_{GE}\sigma_G\sigma_E) \times G_{std} \times E_{std} + \varepsilon' \end{aligned}$$

where ε' is a constant that depends neither on G_{std} nor E_{std}. This leads to the following relationship between the standardized and unstandardized estimates:

$$\beta'_G = \beta_G \times \sigma_G + \beta_{GE} \times \mu_E \times \sigma_G \quad \Leftrightarrow \quad \beta_G = \frac{\beta'_G}{\sigma_G} - \frac{\beta'_{GE} \times \mu_E}{\sigma_E \times \sigma_G}$$

$$\beta'_E = \beta_E \times \sigma_E + \beta_{GE} \times \mu_G \times \sigma_E \quad \Leftrightarrow \quad \beta_E = \frac{\beta'_E}{\sigma_E} - \frac{\beta'_{GE} \times \mu_G}{\sigma_E \times \sigma_G}$$

$$\beta'_{GE} = \beta_{GE} \times \sigma_E \times \sigma_G \quad \Leftrightarrow \quad \beta_{GE} = \frac{\beta'_{GE}}{\sigma_E \times \sigma_G}$$

The variances of the unstandardized estimates equal:

$$\sigma^2_{\beta_G} = var\left(\frac{\beta'_G}{\sigma_G} - \frac{\beta'_{GE} \times \mu_E}{\sigma_E \times \sigma_G}\right) = \frac{\sigma^2_{\beta'_G}}{\sigma^2_G} + \frac{\sigma^2_{\beta'_{GE}} \times \mu^2_E}{\sigma^2_E \times \sigma^2_G} - 2\left(\frac{\mu_E}{\sigma_E \times \sigma^2_G}\right) * cov(\beta'_G, \beta'_{GE})$$

$$\sigma^2_{\beta_E} = var\left(\frac{\beta'_E}{\sigma_E} - \frac{\beta'_{GE} \times \mu_G}{\sigma_E \times \sigma_G}\right) = \frac{\sigma^2_{\beta'_E}}{\sigma^2_E} + \frac{\sigma^2_{\beta'_{GE}} \times \mu^2_G}{\sigma^2_E \times \sigma^2_G} - 2\left(\frac{\mu_G}{\sigma^2_E \times \sigma_G}\right) * cov(\beta'_E, \beta'_{GE})$$

$$\sigma^2_{\beta_{GE}} = var\left(\frac{\beta'_{GE}}{\sigma_E \times \sigma_G}\right) = \frac{\sigma^2_{\beta'_{GE}}}{\sigma^2_E \times \sigma^2_G}$$

Appendix C shows that $cov(\beta'_G, \beta'_{GE}) = cov(\beta'_E, \beta'_{GE}) = 0$ when G and E are independents. Moreover, when G and E are standardized, $\sigma^2_{\beta'_G} = \sigma^2_{\beta'_E} = \sigma^2_{\beta'_{GE}} \approx \frac{\sigma^2}{N}$ when the sample size is large, where σ^2 is the residual variance of Y. Assuming the main effects of G and E and their interaction is small and $\sigma^2 = 1$, the variance of the estimates simplifies:

$$\sigma^2_{\beta_G} \approx \frac{\mu^2_E + \sigma^2_E}{N \times \sigma^2_E \times \sigma^2_G}$$

$$\sigma^2_{\beta_E} \approx \frac{\mu^2_G + \sigma^2_G}{N \times \sigma^2_E \times \sigma^2_G}$$

$$\sigma^2_{\beta_{GE}} \approx \frac{1}{N \times \sigma^2_E \times \sigma^2_G}$$

Appendix B: Non-centrality parameters for marginal and interaction models

Using the estimates and variances from **Appendix A** one can derive ncp_G, ncp_E, and ncp_{GE}, the non-centrality parameters (ncp) of the genetic main effect, the exposure main effect and the interaction effect under the assumptions of small effect sizes and $G-E$ independence:

$$ncp_G = \frac{\beta_G^2}{\sigma_{\beta_G}^2} = \frac{\beta_G^2}{\frac{\mu_E^2+\sigma_E^2}{N\times\sigma_E^2\times\sigma_G^2}} = N\times\sigma_G^2\times\beta_G^2\times\frac{\sigma_E^2}{\mu_E^2+\sigma_E^2}$$

$$ncp_E = \frac{\beta_E^2}{\sigma_{\beta_E}^2} = \frac{\beta_E^2}{\frac{\mu_G^2+\sigma_G^2}{N\times\sigma_G^2\times\sigma_E^2}} = N\times\sigma_E^2\times\beta_E^2\times\frac{\sigma_G^2}{\mu_G^2+\sigma_G^2}$$

$$ncp_{GE} = \frac{\beta_{GE}^2}{\sigma_{\beta_{GE}}^2} = \frac{\beta_{GE}^2}{\frac{1}{N\times\sigma_E^2\times\sigma_G^2}} = N\times\sigma_E^2\times\sigma_G^2\times\beta_{GE}^2$$

These ncp can be compared with ncp_{mG}, the non-centrality parameter from the test of G in a marginal model. The marginal effect of G, β_{mG} is by definition the sum of the main effect of G plus the marginal contribution from interaction terms involving G which equals:

$$\beta_{mG} = \frac{cov(Y,G)}{\sigma_G^2} = \frac{\beta_G'\times\sigma_G}{\sigma_G^2} = \beta_G+\beta_{GE}\times\mu_E$$

The marginal estimated effect of E can be derived similarly and equals:

$$\beta_{mE} = \beta_E+\beta_{GE}\times\mu_G$$

so that ncp_{mG} and ncp_{mE} (the *ncp* of the marginal test of E) can be expressed as follows:

$$ncp_{mG} = N\times\sigma_G^2\times(\beta_G+\beta_{GE}\times\mu_E)^2$$

$$ncp_{mE} = N\times\sigma_E^2\times(\beta_E+\beta_{GE}\times\mu_G)^2$$

Appendix C: Variance-covariance for the GxE term and its estimated effect

To derive the covariance and the correlation parameter between G and $G\times E$ we first calculate σ_{GE}^2, the variance of the interaction term $G\times E$ under the assumption of independence between G and E. Assuming the standard coding for G [0,1,2], and the frequency of the coded allele is p, and E is normally distributed so that E^2 follows a non-central chi-square distribution with one degree of freedom, it can be express as:

$$\sigma_{GE}^2 = \mathbb{E}[G^2]\times\mathbb{E}[E^2]-\mathbb{E}[G]^2\times\mathbb{E}[E]^2 = \sum_{G\in Range(G)}\left(G^2\times pr(G)\right)\times\int_{-\infty}^{+\infty}E^2dP-(2\times p)^2\times\mu_E^2$$

$$= ((2\times p\times(1-p))+p^2\times 4)\times\sigma_E^2\times\left(1+\frac{\mu_E^2}{\sigma_E^2}\right)-(2\times p)^2\times\mu_E^2$$

$$= 2 \times (p + p^2) \times (\sigma_E^2 + \mu_E^2) - 4 \times p^2 \times \mu_E^2$$

$$= \sigma_G^2 \times \sigma_E^2 + \mu_G^2 \times \sigma_E^2 + \mu_E^2 \times \sigma_G^2$$

Under the same assumption, $cov(G, G \times E)$, the covariance between G and $G \times E$ can be expressed as:

$$cov(G, G \times E) = \mathbb{E}[G^2] \times \mathbb{E}[E] - \mathbb{E}[G] \times \mathbb{E}[G] \times \mathbb{E}[E]$$

$$= (2 \times p \times (1 - p) + p^2 \times 4) \times \mu_E - (2 \times p)^2 \times \mu_E$$

$$= \mu_E \times \sigma_G^2$$

Similarly, one can derive the covariance between the exposure and the interaction and show that $cov(E, G \times E) = \mu_G \times \sigma_E^2$. From this it appears that $cov(G, G \times E_{std}) = cov(E, G_{std} \times E) = cov(G_{std}, G_{std} \times E_{std}) = cov(E_{std}, G_{std} \times E_{std}) = 0$.

The correlation between G and $G \times E$ equals then:

$$cor(G, G \times E) = \frac{cov(G, G \times E)}{\sigma_G \times \sigma_{GE}} = \frac{\mu_E \times \sigma_G^2}{\sigma_G \times \sigma_{GE}} = \frac{\mu_E}{\sqrt{\sigma_E^2 \times \left(1 + \frac{\mu_G^2}{\sigma_G^2}\right) + \mu_E^2}}$$

We derive then the covariance and correlation between the estimated effect of G and $G \times E$. In its general form the variance-covariance matrix of estimates from the interaction model can be expressed as: $\sigma_{\boldsymbol{\beta}} = \left(\mathbf{X}^{\mathrm{T}}\mathbf{X}\right)^{-1}\sigma^2$, where $\mathbf{X}$, the matrix of predictor variables, equals $[1, G, E, G \times E]$ and σ^2 is the variance of ε, the residual of Y.

$$\sigma_{\boldsymbol{\beta}} = \left(N \times \begin{bmatrix} \mathbb{E}[1^2] & \mathbb{E}[G] & \mathbb{E}[E] & \mathbb{E}[G \times E] \\ \mathbb{E}[G] & \mathbb{E}[G^2] & \mathbb{E}[G \times E] & \mathbb{E}[G^2 \times \mathrm{E}] \\ \mathbb{E}[E] & \mathbb{E}[G \times E] & \mathbb{E}[E^2] & \mathbb{E}[G \times E^2] \\ \mathbb{E}[G \times E] & \mathbb{E}[G^2 \times \mathrm{E}] & \mathbb{E}[G \times E^2] & \mathbb{E}[G^2 \times E^2] \end{bmatrix} \right)^{-1} \times \sigma^2$$

This is a relatively complex form when G and E are not standardized. However when the predictors are standardized $\mathbb{E}[E] = \mathbb{E}[G] = 0$, and assuming G and E are independents, the formulation of $\sigma_{\boldsymbol{\beta}}$ greatly simplify, as all the off-diagonal elements of the matrix are null, so that:

$$\sigma_{\boldsymbol{\beta}} = \frac{\sigma^2}{N} \begin{bmatrix} 1 & 0 & 0 & 0 \\ 0 & \frac{1}{\mathrm{E}[G^2]} & 0 & 0 \\ 0 & 0 & \frac{1}{\mathrm{E}[E^2]} & 0 \\ 0 & 0 & 0 & \frac{1}{\mathrm{E}[G^2] \times \mathrm{E}[E^2]} \end{bmatrix}$$

which implies that all covariance term, including $cov(\beta'_G, \beta'_{GE})$ are null. Building on this, and using the equations from **Appendix A**, we can derive γ, the covariance between β_G and β_{GE} for the unstandardized case:

$$\gamma = cov(\beta_G, \beta_{GE}) = cov\left(\frac{\beta'_G}{\sigma_G} - \beta_{GE} \times \mu_E, \beta_{GE}\right) = cov\left(\frac{\beta'_G}{\sigma_G}, \frac{\beta'_{GE}}{\sigma_E \times \sigma_G}\right) + \mu_E \times cov(\beta_{GE}, \beta_{GE})$$

$$= \frac{cov(\beta'_G, \beta'_{GE})}{\sigma_E \times \sigma_G^2} - \mu_E \times \sigma^2_{\beta_{GE}} = \frac{-\mu_E}{N \times \sigma_E^2 \times \sigma_G^2}$$

The correlation follows:

$$cor(\beta_G, \beta_{GE}) = \frac{cov(\beta_G, \beta_{GE})}{\sigma_{\beta_G} \times \sigma_{\beta_{GE}}} = \frac{-\mu_E \times \sqrt{\frac{1}{N \times \sigma_E^2 \times \sigma_G^2}}}{\sqrt{\frac{\mu_E^2 + \sigma_E^2}{N \times \sigma_E^2 \times \sigma_G^2}}} = \frac{-\mu_E}{\sqrt{\mu_E^2 + \sigma_E^2}}$$

Appendix D: Derivation of the Pratt index

To estimate the variance explained by predictors or other related measures, we first derive the expected variance of the outcome for a given generative model. For a single interaction term and assuming $G - E$ independence, it equals:

$$var(Y) = var(\beta_E \times E + \beta_G \times G + \beta_{GE} \times G \times E + \varepsilon)$$

$$= \beta_E^2 \times \sigma_E^2 + \beta_G^2 \times \sigma_G^2 + \beta_{GE}^2 \times \sigma_{GE}^2 + 2 \times \beta_{GE} \times \big(\beta_G \times cov(G, GE) + \beta_E \times cov(E, GE)\big) + \sigma^2$$

$$= (\beta_G \times \sigma_G + \beta_{GE} \times \mu_E \times \sigma_G)^2 + (\beta_E \times \sigma_E + \beta_{GE} \times \mu_G \times \sigma_E)^2 + (\beta_{GE} \times \sigma_E \times \sigma_G)^2 + \sigma^2$$

$$= \beta_G'^2 + \beta_E'^2 + \beta_{GE}'^2 + \sigma^2$$

When more interaction terms are included, the outcome variance becomes a little more complex as additional covariance terms are added. For example assuming k interactions between E and G_i, $i = 1 \ldots k$, the variance of Y becomes:

$$var(Y) = \beta_E^2 \times \sigma_E^2 + \sum_i \left[\beta_{G_i}^2 \times \sigma_{G_i}^2\right] + \sum_i \left[\beta_{G_iE}^2 \times \sigma_{G_iE}^2\right] + 2 \times \sum_i \left[\beta_{G_iE} \times \beta_{G_i} \times \mu_E \times \sigma_{G_i}^2\right] + 2 \times \sum_i \beta_{G_iE} \times \beta_E \times \mu_{G_i} \times \sigma_E^2 + \sum_i \sum_{j \neq i} \left[cov\big(G_i \times E, G_j \times E\big)\right] + \sigma^2$$

$$= \beta_E^2 \times \sigma_E^2 + \sum_i \left[\beta_{G_i}^2 \times \sigma_{G_i}^2\right] + \sum_i \left[\beta_{G_iE}^2 \times \sigma_{G_iE}^2\right] + 2 \times \sum_i \left[\beta_{G_iE} \times \beta_{G_i} \times \mu_E \times \sigma_{G_i}^2\right] + 2 \times \sum_i \beta_{G_iE} \times \beta_E \times \mu_{G_i} \times \sigma_E^2 + \sum_i \sum_{j \neq i} \left[\beta_{G_iE} \times \beta_{G_jE} \times \mu_{G_i} \times \mu_{G_j} \times \sigma_E^2\right] + \sigma^2$$

For simplicity let us assume σ^2 is set so that $var(Y) = 1$ in all further derivation. When testing a single interaction term and using the equivalences from **Appendix A-B** , it follows that the Pratt index can be expressed as a function of the estimates from the interaction model and the mean and variance of the genetic variant and the exposures considered:

$$r_G^{2*} = (\beta_G \times \sigma_G) \times cor(Y, G) = (\beta_G \times \sigma_G) \times \beta_{mG} \times \sigma_G = \beta_G^2 \times \sigma_G^2 + \beta_G \times \beta_{GE} \times \sigma_G^2 \times \mu_E$$

$$r_E^{2*} = (\beta_E \times \sigma_E) \times cor(Y, E) = (\beta_E \times \sigma_E) \times (\beta_E + \beta_{GE} \times \mu_G) \times \sigma_E = \beta_E^2 \times \sigma_E^2 + \beta_E \times \beta_{GE} \times \sigma_E^2 \times \mu_G$$

$$r_{GE}^{2*} = (\beta_{GE} \times \sigma_{GE}) \times cor(Y, G \times E)$$

$$= (\beta_{GE} \times \sigma_{GE}) \times \frac{cov(\beta_G \times G + \beta_E \times E + \beta_{GE} \times G \times E + \varepsilon, G \times E)}{\sigma_{GE}}$$

$$= \beta_{GE} \times (\beta_G \times \mu_E \times \sigma_G^2 + \beta_E \times \mu_G \times \sigma_E^2 + \beta_{GE} \times \sigma_{GE}^2)$$

$$= \beta_{GE}^2 \times (\mu_G^2 \times \sigma_E^2 + \mu_E^2 \times \sigma_G^2 + \sigma_E^2 \times \sigma_G^2) + \beta_{GE} \times (\beta_G \times \mu_E \times \sigma_G^2 + \beta_E \times \mu_G \times \sigma_E^2)$$

When summing the above Pratt index we obtain:

$$r_G^{2*} + r_E^{2*} + r_{GE}^{2*} = \beta_G^2 \times \sigma_G^2 + \beta_G \times \beta_{GE} \times \sigma_G^2 \times \mu_E + \beta_E^2 \times \sigma_E^2 + \beta_E \times \beta_{GE} \times \sigma_E^2 \times \mu_G + \beta_{GE}^2 \times \mu_G^2 \times \sigma_E^2 + \beta_{GE}^2 \times \mu_E^2 \times \sigma_G^2 + \beta_{GE}^2 \times \sigma_E^2 \times \sigma_G^2 + \beta_{GE} \times (\beta_G \times \mu_E \times \sigma_G^2 + \beta_E \times \mu_G \times \sigma_E^2)$$

$$= \beta_G^2 \times \sigma_G^2 + 2 \times (\beta_G \times \sigma_G) \times (\beta_{GE} \times \mu_E \times \sigma_G) + (\beta_{GE} \times \mu_E \times \sigma_G)^2 + \beta_E^2 \times \sigma_E^2 + 2 \times (\beta_E \times \sigma_E) \times (\beta_{GE} \times \mu_G \times \sigma_E) + (\beta_{GE} \times \mu_G \times \sigma_E)^2 + (\beta_{GE} \times \sigma_E \times \sigma_G)^2$$

$$= (\beta_G \times \sigma_G + \beta_{GE} \times \mu_E \times \sigma_G)^2 + (\beta_E \times \sigma_E + \beta_{GE} \times \mu_G \times \sigma_E)^2 + (\beta_{GE} \times \sigma_E \times \sigma_G)^2$$

$$= r_G^2 + r_E^2 + r_{GE}^2$$

The cumulative contribution of multiple interactions involving independent SNPS can also be derived from summary statistics, although the derivation is a little less friendly because of additional covariance terms. For example assuming k interactions between E and G_i, $i = 1 \dots k$, we obtain (**Figure S4**) :

$$r_G^{2*} = \sum_i [\beta_{G_i}^2 \times \sigma_{G_i}^2 + \beta_{G_i} \times \beta_{G_iE} \times \sigma_{G_i}^2 \times \mu_E]$$

$$r_E^{2*} = \beta_E^2 \times \sigma_E^2 + \sum_i [\beta_E \times \beta_{G_iE} \times \sigma_E^2 \times \mu_{G_i}]$$

$$r_{GE}^{2*} = \sum_i [\beta_{G_iE}^2 \times \sigma_{G_iE}^2] + \sum_i [\beta_{G_iE} \times \beta_{G_i} \times \mu_E \times \sigma_{G_i}^2] + \sum_i [\beta_{G_iE} \times \beta_E \times \mu_{G_i} \times \sigma_E^2] + \sum_i \left[\beta_{G_iE} \times \sum_{j \neq i} [\beta_{G_jE} \times \mu_{G_i} \times \mu_{G_j} \times \sigma_E^2]\right]$$

On should note that estimating the Pratt index for the exposure can be difficult in practice when the number of interaction is large, as it would require the estimated main exposure effect from a joint model including all SNPs main effect and all interactions term with the exposure. Also, because of the correlation between main and interaction terms, $r_{X_i}^{2*}$, as the standard $r_{X_i}^2$, only approximate the amount the variance will change if X_i was held constant. For the latter measure, one can refer to[21].

The special case of negative Pratt index:

As opposed to standard derivation of the variance explained, the Pratt index formula allows r_X^{2*} being negative. This characteristic has been a source of concerns [Thomas, et al. 1998]. While in-depth discussion of this characteristic is out of the scope of this study, we consider a hypothetical example illustrating this phenomenon.

As negative values for the Pratt index can exist in the presence of correlated predictors (whether or not there interaction effect are modelled), we considered the simplest example of two highly correlated factors A and B (e.g. A being coffee drinking and B being smoking) that define a quantitative outcome Y through the linear model: $Y = \beta_A A - \beta_B B$. We assumed A and B are normal with have variance 1, $cor(A, B) = 0.8$, and $\beta_A = 2\beta_B = 2$. It follows that the variance of Y equals $r_y^2 = \beta_A^2 + \beta_B^2 - 2\beta_A\beta_B cor(A, B) = 1.8$. Using standard approach we would conclude that A and B explain 80% and 20% of the variance of Y. Thus if B was removed from the population (e.g. if everyone in the population stop smoking), a naïve interpretation would be that the variance of Y would decrease according to the amount of variance explain by B. However, if B is removed from the population, then $Y = \beta_A A$ and its variance, $r_{y-B}^2 = \beta_A^2 = 4$ and is therefore larger than before. The Pratt index captures such effects by assigning negative value to some predictors. In this specific case, we would have $r_A^{2*} = 2.4$ and $r_B^{2*} = -0.6$, with the later parameter highlighting the potential increase in variance of Y if B is removed from the population.

Appendix F: GRS-based test, joint test and univariate test of multiple interaction effects

We denote $\boldsymbol{\beta} = (\beta_{G_1}, \beta_{G_2}, \dots \beta_{G_m})$ a vector of effects from m independent SNP, and $\sigma_{\beta_{G_i}}^2$ and w_i are the variance of each estimate and weight of each SNP i in the genetic risk score (GRS), respectively. The effect of the weighted GRS on the outcome, γ_{GRS}, equals:

$$\gamma_{GRS} = \frac{cov(Y, \mathrm{GRS})}{\sigma_{GRS}^2} = \frac{cov(Y, \sum_m [w_i \times G_i])}{\sigma_{\sum_m [w_i \times G_i]}^2} = \frac{\sum_m (w_i \times \beta_{G_i} \times \sigma_{G_i}^2)}{\sum_m [w_i^2 \times \sigma_{G_i}^2]} = \frac{\sum_m \frac{w_i \times \beta_{G_i}}{\sigma_{\beta_{G_i}}^2}}{\sum_m \frac{w_i^2}{\sigma_{\beta_{G_i}}^2}}$$

Consecutively, $\sigma_{\gamma_{GRS}}$ the variance of γ_{GRS} can be derived as follows:

$$\sigma_{\gamma_{GRS}}^2 = var\left(\frac{\sum_m \frac{w_i \times \beta_{G_i}}{\sigma_{\beta_{G_i}}^2}}{\sum_m \frac{w_i^2}{\sigma_{\beta_{G_i}}^2}}\right) = \sum_m \left(\left(\frac{\frac{w_i}{\sigma_{\beta_{G_i}}^2}}{\sum_m \frac{w_i^2}{\sigma_{\beta_{G_i}}^2}}\right)^2 \times var(\beta_{G_i})\right) = \frac{\sum_m \left(\frac{w_i^2}{\sigma_{\beta_{G_i}}^2}\right)}{\left(\sum_m \frac{w_i^2}{\sigma_{\beta_{G_i}}^2}\right)^2} = \frac{1}{\sum_m \frac{w_i^2}{\sigma_{\beta_{G_i}}^2}}$$

So that the chi-square of the marginal effect of the test of GRS equals:

$$\left(\frac{\gamma_{GRS}}{\sigma_{\gamma_{GRS}}}\right)^2 = \frac{\left(\sum_m \frac{w_i \times \beta_{G_i}}{\sigma_{\beta_{G_i}}^2}\right)^2}{\sum_m \frac{w_i^2}{\sigma_{\beta_{G_i}}^2}}$$

which corresponds to the inverse-variance weighted sum meta-analysis of each individual genetic variant. Similarly, one can derive the expected chi-square of the GRS by exposure interaction effect using $\gamma_{G_i \times E}$, the interaction effect between each SNP i and the exposure. Under the assumption of independence of the m interaction terms we obtain:

$$\left(\frac{\gamma_{INT}}{\sigma_{\gamma_{INT}}}\right)^2 = \frac{\left(\sum_m \frac{\gamma_{G_i\times E}}{\sigma^2_{\gamma_{G_i\times E}}}\right)^2}{\sum_m \frac{1}{\sigma^2_{\gamma_{G_i\times E}}}}$$

Hence for standardized G and E the *ncp* of the *GRS* by E interaction test equals $ncp_{\mathrm{GRS\times E}} = N \times \left(\sum_{\mathrm{i=1...m}} \gamma'_{G_i\times E}\right)^2/m$, where *N* is the sample size and $\gamma'_{G_i\times E}$ is the interaction effect from the standardized model, and follows a chi-square with one degree of freedom. In comparison, the *ncp* for the test of the strongest pairwise interaction, i.e. the interaction that explained the largest amount of variance, equals $ncp_{\mathrm{pairwise}} = \max\left(N \times \gamma'_{G_i\times E}\right)$.

Appendix E: Joint test of main and interaction effects

The multiple regression least square provides the estimated effect of the genetic main effect and interaction effects $\boldsymbol{\beta} = (\beta_G, \beta_{GE})$ and their variance-covariance matrix $\boldsymbol{\Sigma}$**.** The multivariate Wald test of the two parameters, which follow a 2 *df* chi-square can be expressed as:

$$\boldsymbol{\beta}^T\boldsymbol{\Sigma}^{-1}\boldsymbol{\beta} = [\beta_G \quad \beta_{GE}]\begin{bmatrix}\sigma^2_{\beta_G} & \gamma \\ \gamma & \sigma^2_{\beta_{GE}}\end{bmatrix}^{-1}\begin{bmatrix}\beta_G \\ \beta_{GE}\end{bmatrix},$$

where γ is the covariance between β_G and β_{GE}. It can be further developed as:

$$\boldsymbol{\beta}^T\boldsymbol{\Sigma}^{-1}\boldsymbol{\beta} = [\beta_G \quad \beta_{GE}] \times \frac{1}{\sigma^2_{\beta_G} \times \sigma^2_{\beta_{GE}} - \gamma^2}\begin{bmatrix}\sigma^2_{\beta_{GE}} & -\gamma \\ -\gamma & \sigma^2_{\beta_G}\end{bmatrix}\begin{bmatrix}\beta_G \\ \beta_{GE}\end{bmatrix}$$

$$= \frac{1}{\sigma^2_{\beta_G} \times \sigma^2_{\beta_{GE}} - \gamma^2} \times \left[\sigma^2_{\beta_{GE}} \times \beta_G - \gamma \times \beta_{GE} \quad -\gamma \times \beta_G + \sigma^2_{\beta_G} \times \beta_{GE}\right]\begin{bmatrix}\beta_G \\ \beta_{GE}\end{bmatrix}$$

$$= \frac{\left(\sigma^2_{\beta_{GE}} \times \beta_G - \gamma \times \beta_{GE}\right) \times \beta_G + \left(\sigma^2_{\beta_G} \times \beta_{GE} - \gamma \times \beta_G\right) \times \beta_{GE}}{\sigma^2_{\beta_G} \times \sigma^2_{\beta_{GE}} - \gamma^2}$$

$$= \frac{\sigma^2_{\beta_{GE}} \times \beta^2_G + \sigma^2_{\beta_G} \times \beta^2_{GE} - 2 \times \gamma \times \beta_G \times \beta_{GE}}{\sigma^2_{\beta_G} \times \sigma^2_{\beta_{GE}} - \gamma^2}$$

For clarity we derived the nominator and the denominator separately, so that $\boldsymbol{\beta}^T\boldsymbol{\Sigma}^{-1}\boldsymbol{\beta} = A/B$

$$A = \hat{\sigma}^2_{\beta_{GE}} \times \left(\frac{\beta'_G}{\sigma_G} - \frac{\beta'_{GE} \times \mu_E}{\sigma_E \times \sigma_G}\right)^2 + \hat{\sigma}^2_{\beta_G} \times \left(\frac{\beta'_{GE}}{\sigma_E \times \sigma_G}\right)^2 + 2 \times \mu_E \sigma^2_{\beta_{GE}} \times \left(\frac{\beta'_G}{\sigma_G} - \frac{\beta'_{GE} \times \mu_E}{\sigma_E \times \sigma_G}\right) \times \left(\frac{\beta'_{GE}}{\sigma_E \times \sigma_G}\right)$$

$$= \hat{\sigma}^2_{\beta_{GE}} \times \left(\left(\frac{\beta'_G}{\sigma_G}\right)^2 - \left(\frac{\beta'_{GE} \times \mu_E}{\sigma_E \times \sigma_G}\right)^2\right) + \hat{\sigma}^2_{\beta_G} \times \left(\frac{\beta'_{GE}}{\sigma_E \times \sigma_G}\right)^2$$

$$= \frac{\left(\frac{\beta'_G}{\sigma_G}\right)^2 - \left(\frac{\beta'_{GE} \times \mu_E}{\sigma_E \times \sigma_G}\right)^2 + \left(\frac{\beta'_{GE} \times \mu_E}{\sigma_E \times \sigma_G}\right)^2 + \left(\frac{\beta'_{GE} \times \sigma_E}{\sigma_E \times \sigma_G}\right)^2}{N \times \sigma^2_E \times \sigma^2_G}$$

$$= \frac{\left(\frac{\beta'_G}{\sigma_G}\right)^2 + \left(\frac{\beta'_{GE}}{\sigma_G}\right)^2}{N \times \sigma_E^2 \times \sigma_G^2}$$

The denominator B equals:

$$B = \hat{\sigma}_{\beta_G}^2 \times \hat{\sigma}_{\beta_{GE}}^2 - \left(-\mu_E \times \sigma_{\beta_{GE}}^2\right)^2 = \frac{\sigma_E^2}{(N \times \sigma_E^2 \times \sigma_G^2)^2}$$

So that the joint test of G and $G \times E$ effects equals:

$$\boldsymbol{\beta}^T \boldsymbol{\Sigma}^{-1} \boldsymbol{\beta} = N \times \sigma_E^2 \times \frac{\left(\frac{\beta'_G}{\sigma_G}\right)^2 + \left(\frac{\beta'_{GE}}{\sigma_G}\right)^2}{\sigma_E^2 \times \sigma_G^2} = N \times \beta_G'^2 + N \times \beta_{GE}'^2$$

which is the sum of the individuals Wald test for the main effect and the interaction effect when G and E are standardized. Moreover, leveraging previous equivalences, we can express the joint test as a function of β''_G and β''^2_{GE}, the estimated main and interaction effects from the model where E has been centered, it follows that:

$$\boldsymbol{\beta}^T \boldsymbol{\Sigma}^{-1} \boldsymbol{\beta} = N \times \beta_G''^2 \times \sigma_G^2 + N \times \beta_{GE}''^2 \times \sigma_G^2 \times \sigma_E^2$$

Figure S1. Linear interaction effect across different coding schemes

Pattern of contribution of an interaction term to an outcome when using the standard coding ([0,1,2], upper panel) or using centered values ([-1,0,1], lower panel) for the genotype, and using a normal distribution for the exposure with variance 1 and mean of 0 (a,e), 1 (b,f), 5 (c,g) and 10 (d,h) in the generative model. The interaction effects were set so that they explain 1% of the outcome across all models.

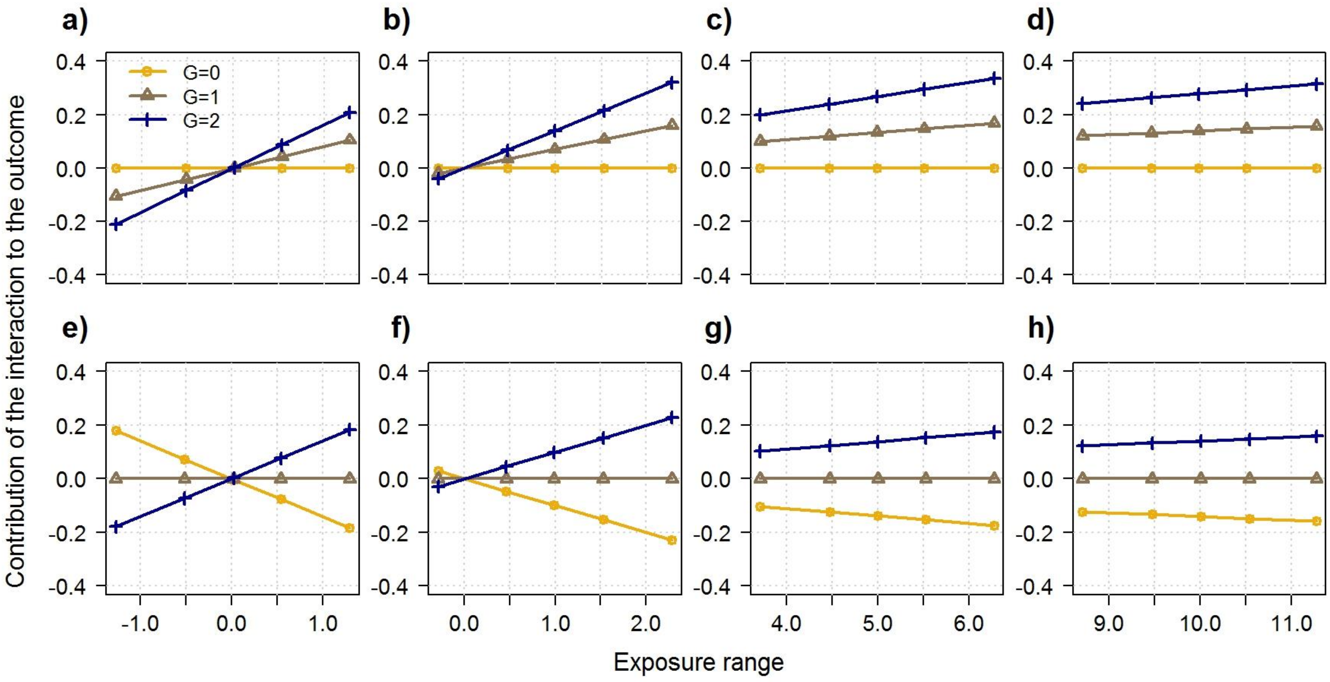

Figure S2. Power comparison for linear regression

A normally distributed outcome Y is generated as a function of the main effect of a genetic variants G, the main effect of an exposure E and an interaction effect between G and E, using four different distributions of the exposure (lower panel). In scenario a) E is normally distributed with mean 0 and variance 1. In scenario b) E follows an exponential distribution with lambda parameter 1, so that the mean and the variance equal 1. In scenario *c*) E follows a uniform distribution with minimum 0 and maximum 10. In scenario d) E is normally distributed with mean 4 and variance 1. Power of the test of marginal genetic effect (marg.G), the main genetic effect (main.G), the interaction effect (int.GxE) and the joint test of main and interaction effect (Joint.G.GxE) are derived for each scenario at the significance level of $5x10^{-8}$ across 1,000 simulations each including 20,000 samples. The contribution of E to the variance of Y, v_E, equals 1%, while the contribution of G, v_G, is either null (upper panels) or equals to v_{GE}, the contribution of the interaction term (middle panel). The v_{GE} are set so that the joint test achieve 60% power on average. In all scenarios where the mean of the exposure is large as compared to its standard deviation, the joint test and the marginal test of G have dramatically larger power as compared to the interaction test, even when the underlying model includes an interaction effect only.

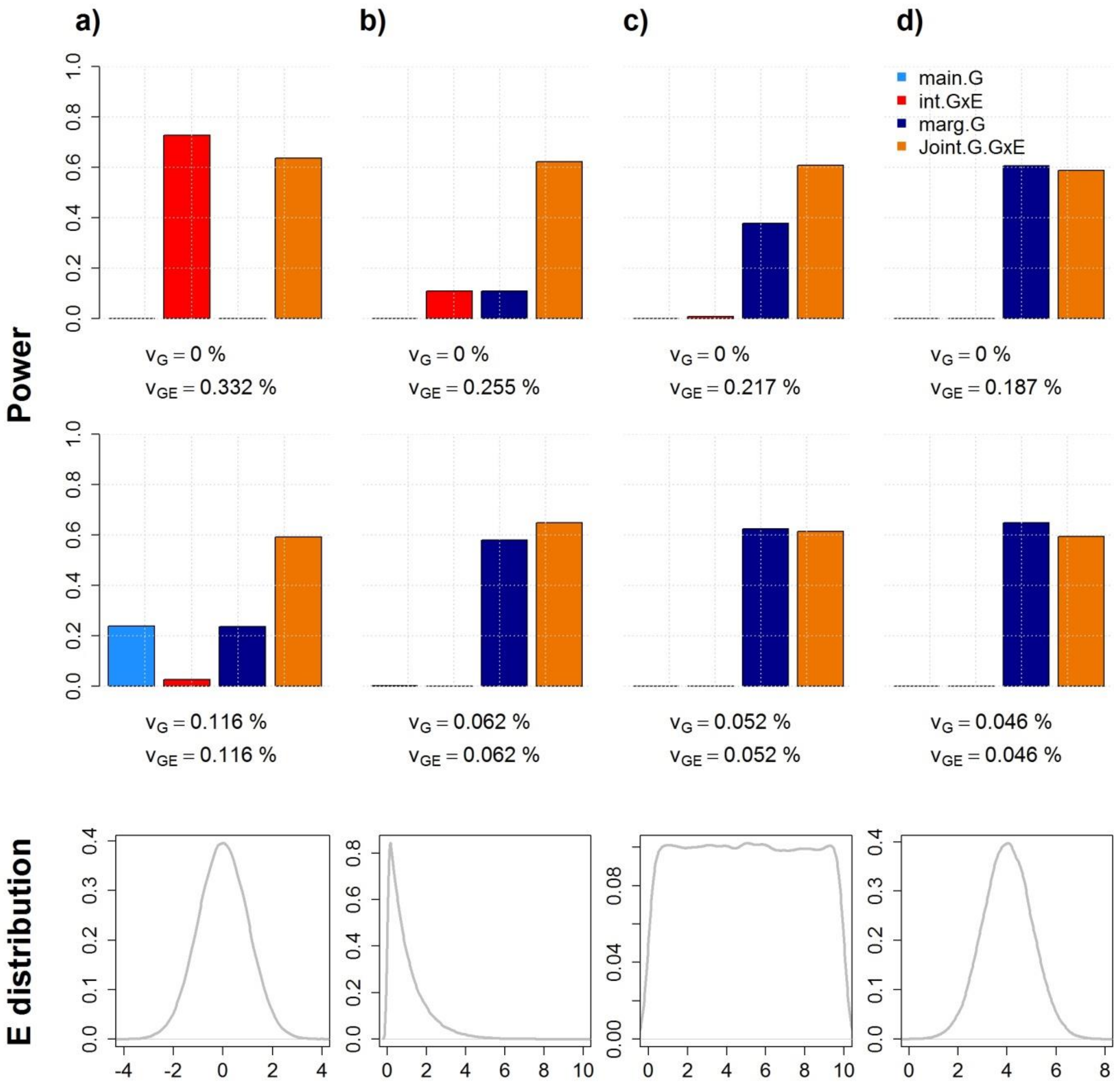

Figure S3. Power comparison for logistic regression

A binary outcome *Y* with a prevalence of 30% is generated as a function of the main effect of a genetic variants *G*, the main effect of an exposure *E* and an interaction effect between *G* and *E*, using four different distributions of the exposure (lower panel). In scenario a) *E* is normally distributed with mean 0 and variance 1. In scenario b) *E* follows an exponential distribution with lambda parameter 1, so that the mean and the variance are equal 1. In scenario *c*) *E* is normally distributed with mean 4 and variance 1. In scenario d) *E* follows a uniform distribution with minimum 0 and maximum 10. Power of the test of marginal genetic effect (marg.G), the main genetic effect (main.G), the interaction effect (int.GxE) and the joint test of main and interaction effect (Joint.G.GxE) is derived for each scenario at the significance level of $5x10^{-8}$ across 1,000 simulations each including 20,000 samples. The exposure has an odds ratio of 1.1, while the odds ratio of *G* and *GxE* are set so that the joint test achieve 60% power on average. In all scenarios where the mean of the exposure is large as compared to its standard deviation, the joint test and the marginal test of G have dramatically larger power as compared to the interaction test, even when the underlying model includes an interaction effect only.

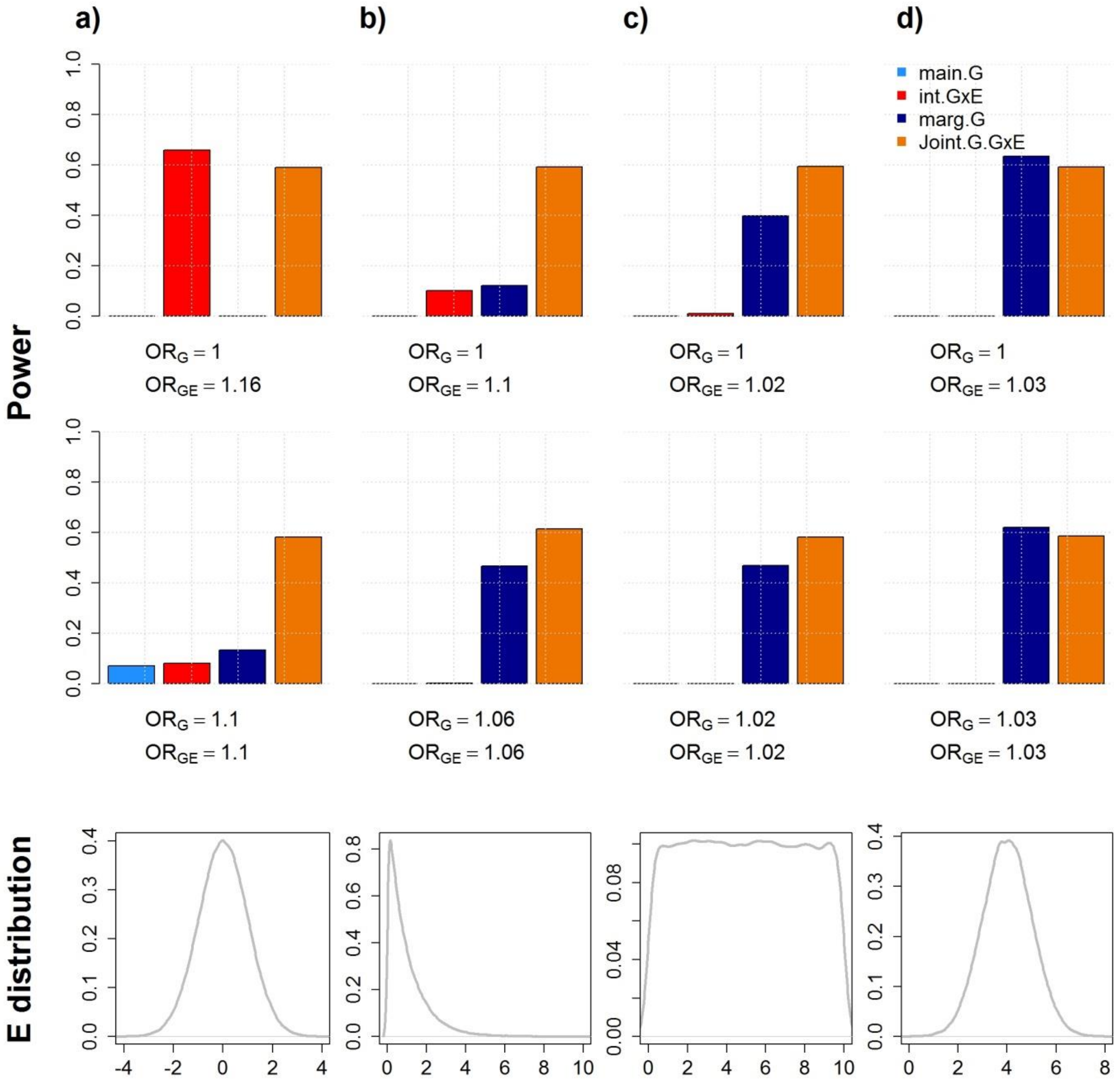

Figure S4. The Pratt index across multiple interactions.

Examples of the cumulated contribution of the genetic, exposure and interaction effects to the variance of an outcome Y when using the standard approach (variance explained by the interaction term on top of the marginal effect, $\sum r^2$) and the Pratt index ($\sum r^{*2}$). The outcome was generated as a function of 10,000 independent causal SNPs and a single exposure. In scenario a) the exposure is binary and rare (5% prevalence) and modifies the effect of 10% of the causal SNPS. In scenario b) the exposure is binary and very common (90% prevalence) and modifies the effect of 30% of the causal SNPs. In scenario c) the exposure is normally distributed with mean 4 and variance 1 and modifies the effect of 60% of the causal SNPs. The frequencies of the causal SNPs are drawn from a uniform distribution with a minimum of 0.01 and a maximum of 0.99. For simplicity all main genetic effect and interaction effects have equal effect size and the direction of the interaction effects are set so the average contribution of interaction effects to the marginal effect of the exposure is null. The genetic main effects and interaction effects combined explain 60% of the variance of Y, and the exposure main effect explains 10%. The standard approach shows a similar pattern across the 3 scenarios, while the Pratt index correctly recovers the cumulated contribution of the GxE terms (i.e. as defined in the generative model).

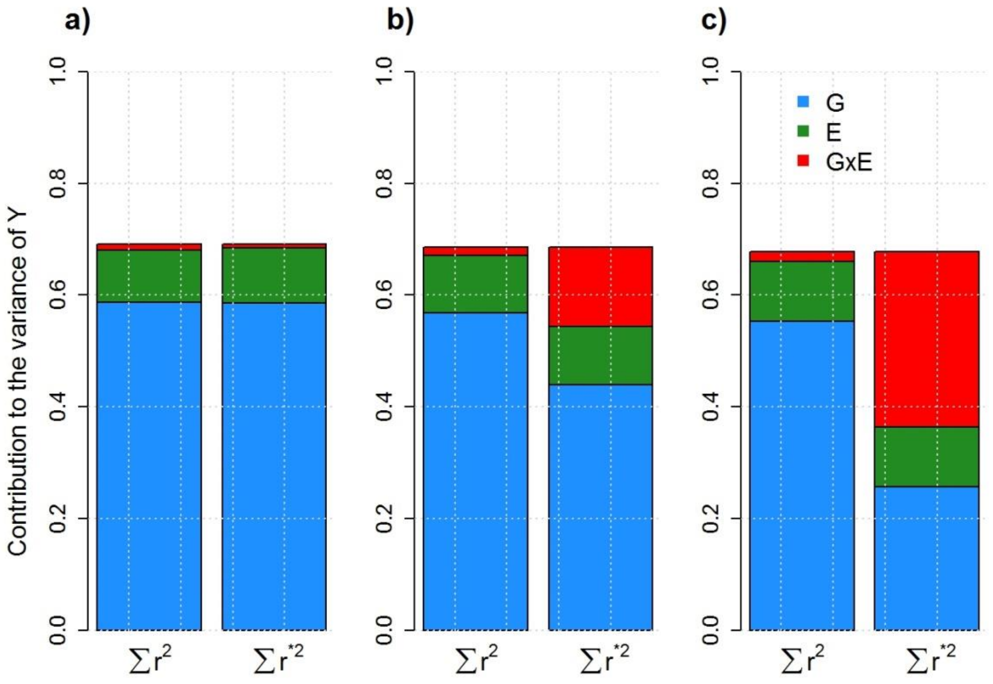

Figure S5. Joint test of main genetic effect and multiple interaction effects in a linear regression.

A normally distributed outcome Y, a genetic variants G and 10 non-centered normally distributed exposures $\mathbf{E} = (E_1, E_2, \dots, E_{10})$ are generated for 2,000 individuals across 100,000 replicates. The distribution of the -log10(*p*-value) of two tests for the joint analysis of the main genetic effect and the 10 interaction effects between G and $\mathbf{E}$ are compared under a null model of no main genetic effect and no interaction: the multivariate Wald test of estimates obtained from the interaction model when using the raw exposures (upper panels, blue), and a test based on the sum of chi-squares from individuals main and interaction estimates obtain from the same model but after centering the exposures (middle panels, red). The correlation between these two tests is then compared under an alternative hypothesis where the variance explained by the main genetic effect and the interaction terms are drawn from a uniform [0, 0.002] (bottom panels). Four scenarios are considered: in a) $\mathbf{E}$ is multivariate normal with no correlation between the exposures; in b) $\mathbf{E}$ is multivariate normal with an average absolute pairwise correlation of 0.07 between the exposures (95% of the correlations are in [-0.19, 0.19]); in c) $\mathbf{E}$ is multivariate normal with an average absolute pairwise correlation of 0.22 between the exposure (95% of the correlations are in [-0.44, 0.44]); and in d) $\mathbf{E}$ is multivariate log-normal with no correlation between the exposures. Three patterns are considered for the genetic variant, i) *G* is not associated with $\mathbf{E}$ (*G₀*); ii) *G* is causal for 3 out of the 10 exposures with effect size (i.e. the exposure's variance explained) drawn from a univariate [0, 0.01] (G_1); and iii) *G* is causal for 3 out of the 10 exposures with effect size drawn from a univariate [0, 0.05] (G_2).

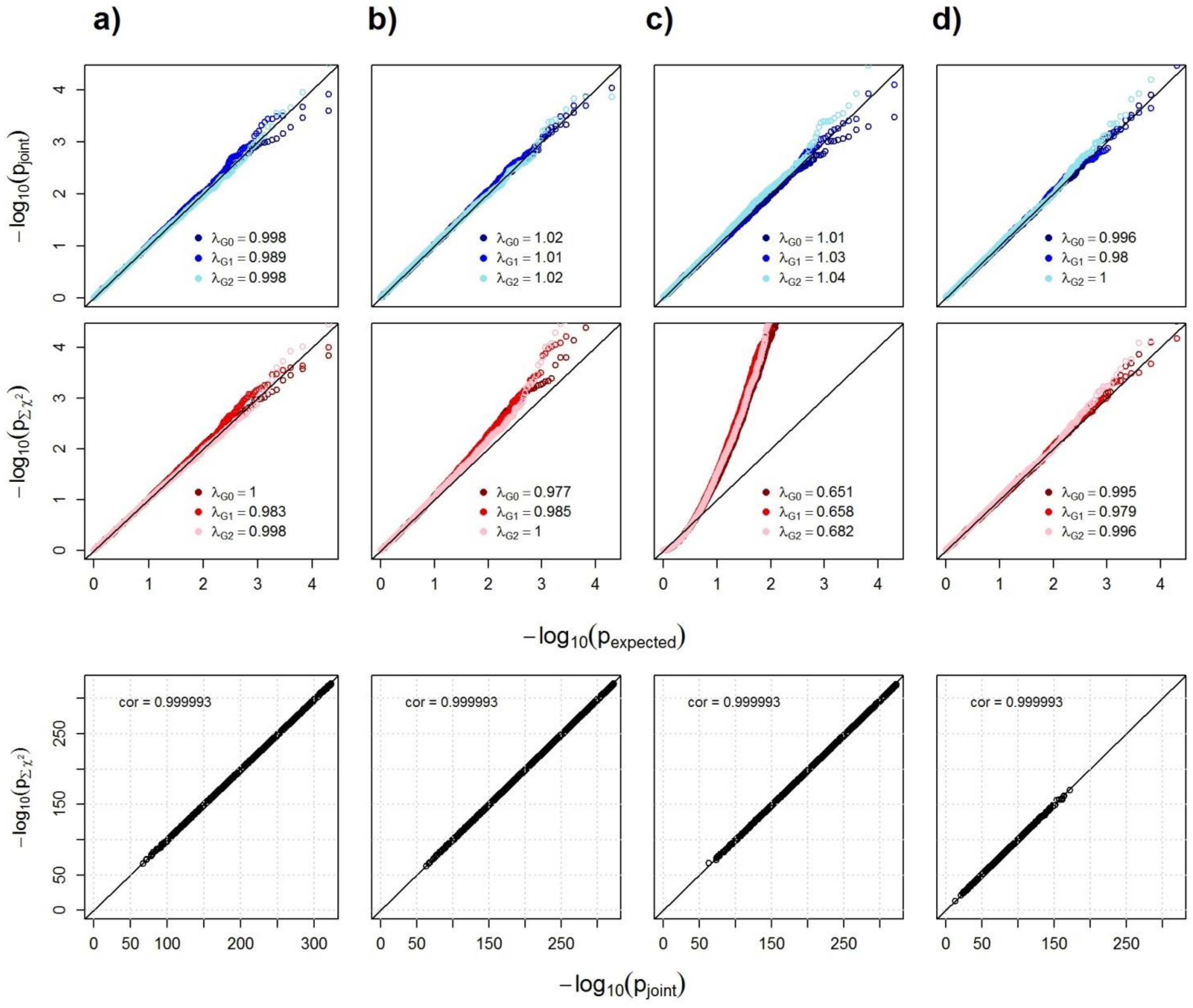

Figure S6. Joint test of main genetic effect and multiple interaction effects in a logistic regression.

A binary outcome Y with 40% prevalence, a genetic variant G and 10 non-centered normally distributed exposures $\mathbf{E} = (E_1, E_2, \ldots, E_{10})$ are generated for 2,000 individuals across 100,000 replicates. The distribution of the -log10(*p*-value) of two tests for the joint analysis of the main genetic effect and the 10 interaction effects between G and $\mathbf{E}$ are compared under a null model of no main genetic effect and no interaction: the multivariate Wald test of estimates obtained from the interaction model when using the raw exposures (upper panels, blue), and a test based on the sum of chi-squares from individuals main and interaction estimates obtain from the same model but after centering the exposures (middle panels, red). The correlation between these two tests is then compared under an alternative hypothesis where the main genetic effect and the interaction effect have an odds ratio of 1.1 (bottom panels). Four scenarios are considered: in a) $\mathbf{E}$ is multivariate normal with no correlation between the exposures; in b) $\mathbf{E}$ is multivariate normal with an average absolute pairwise correlation of 0.07 between the exposures (95% of the correlations are in [-0.19, 0.19]); in c) $\mathbf{E}$ is multivariate normal with an average absolute pairwise correlation of 0.22 between the exposure (95% of the correlations are in [-0.44, 0.44]); and in d) $\mathbf{E}$ is multivariate log-normal with no correlation between the exposures. Three patterns are considered for the genetic variant, i) G is not associated with $\mathbf{E}$ (G_0); ii) G is causal for 3 out of the 10 exposures with effect size (i.e. the exposure's variance explained) drawn from a univariate [0, 0.01] (G_1); and iii) G is causal for 3 out of the 10 exposures with effect size drawn from a univariate [0, 0.05] (G_2).

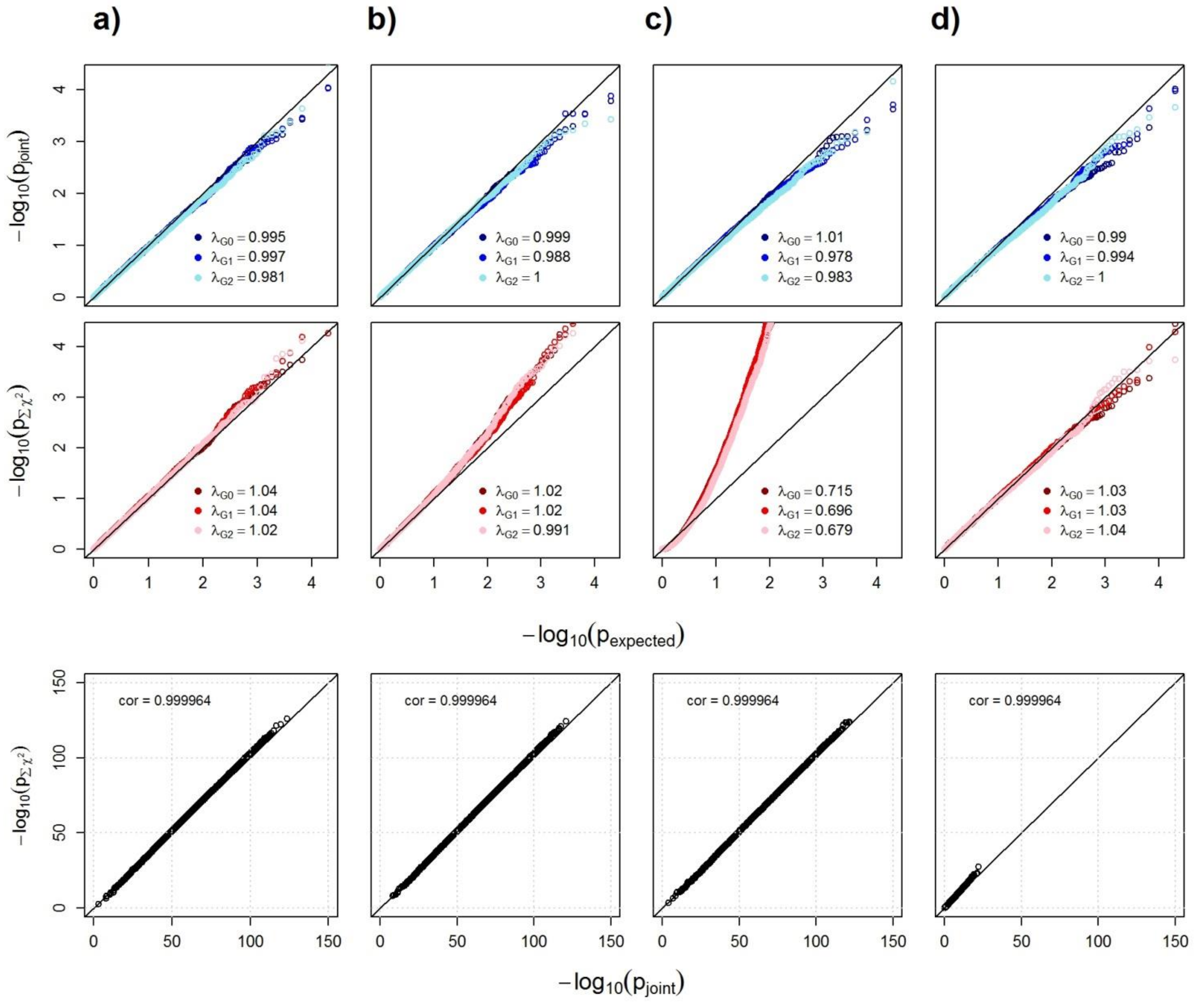

Figure S7. GRS-based statistic and meta-analysis of single SNP estimates in linear regression.

A normally distributed outcome Y is generated as a function of the main effect of 20 genetic variants, the main effect of an exposure E and 20 interaction effects between the exposure and each of the 20 genetic variants across 1,000 replicates including each 2,000 samples. Three distributions of the exposure are considered. In scenario a) E is normally distributed with mean 3 and variance 1; in scenario b) E follows a uniform distribution with minimum 0 and maximum 10; and in scenario c) E follows an exponential distribution with parameter lambda of 1. The GRS-based test of marginal genetic effect ($Y \sim GRS$, left panels), and main (center panels) and interaction (right panels) effects from the interaction model ($Y \sim GRS + E + GRS \times E$) are compared against the corresponding inverse-variance weighted meta-analysis of univariate statistics.

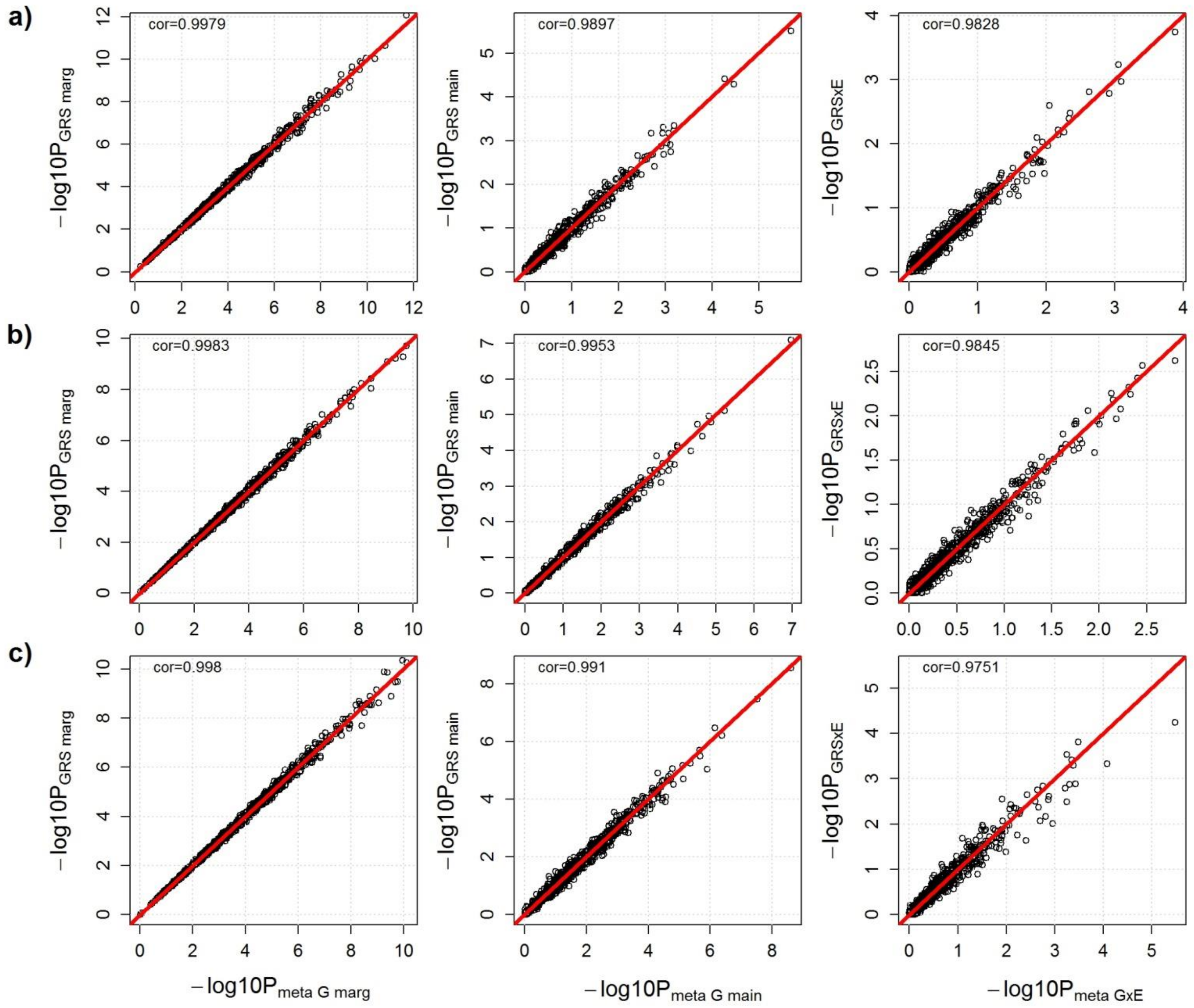

Figure S8. GRS-based statistic and meta-analysis of single SNP estimates in logistic regression.

A binary outcome Y with prevalence of 40% is generated as a function of the main effect of 20 genetic variants, the main effect of an exposure E and 20 interaction effects between the exposure and each of the 20 genetic variants across 1,000 simulations including each 2,000 samples. Three distributions of the exposure are considered. In scenario a) E is normally distributed with mean 3 and variance 1; in scenario b) E follows a uniform distribution with minimum 0 and maximum 10; and in scenario c) E follows an exponential distribution with parameter lambda of 1. The GRS-based test of marginal genetic effect ($logit(Pr_{Y=1}) \sim GRS$, left panels), and main (center panels) and interaction (right panels) effect from the interaction model ($logit(Pr_{Y=1}) \sim GRS + E + GRS \times E$) are compared against the corresponding inverse-variance weighted meta-analysis of univariate statistics.

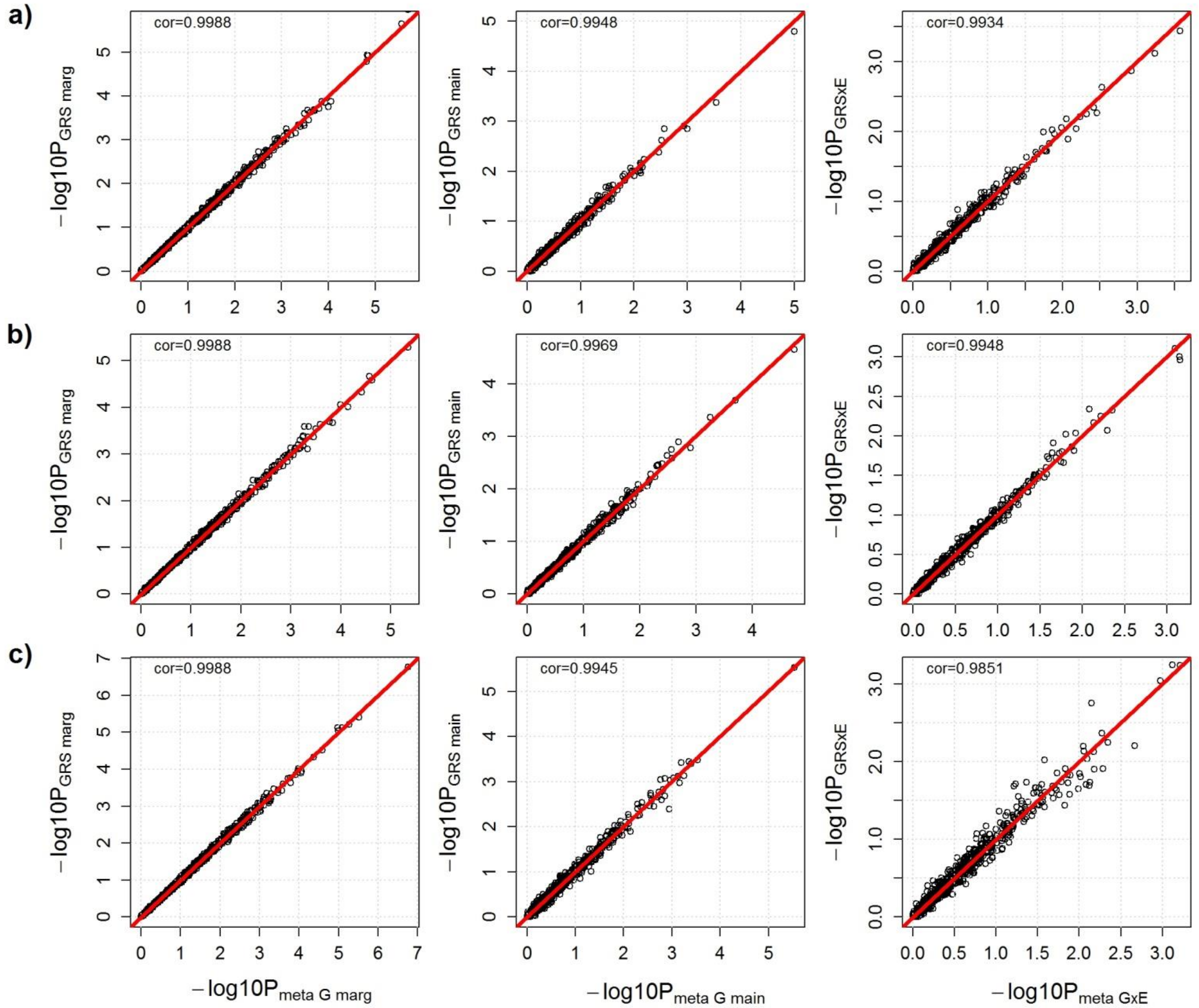

Table S1. Variable and effect estimates for SNPs by Soda consumption interaction on BMI

Variable	Effect estimates		Variable distribution	
	Marginal model	***Interaction model***	***Mean***	***Variance***
GRS	1.212	0.683	2.900	0.260
Soda consumption	-0.134	-0.879	2.062	1.078
Interaction	-	0.257	-	-

Derivation was done using the Nurses' Health Study (NHS) and Health Professional Follow-up Study (HPFS) data only.

Table S2. Variable and effect estimates for SNPs by physical activity interaction on BMI

Variable	Effect estimates		Variable distribution	
	Marginal model	***Interaction model***	***Mean***	***Variance***
GRS	0.161	0.192	11.20	4.84
Physical Activity	-0.358	-0.212	2.50*	1.25*
Interaction	-	-0.013	-	-

* *physical activity was defined following the Cambridge Physical Activity Index in four levels (1= inactive, 2= moderate inactive, 3= moderate active and 4= active). Mean and variance of the exposure was not available in the study and was approximated assuming equal probability across the four groups.*
